# Highly Luminescent Nucleoside-Based N, P-Doped Carbon Dots for Sensitive Detection of Ions and Bioimaging

**DOI:** 10.3389/fchem.2022.906806

**Published:** 2022-06-07

**Authors:** Mengru Wang, Mengling Liu, Shuli Nong, Wenzhu Song, Xianpeng Zhang, Shuang Shen, Guohong Jian, Xiangyao Chen, Zhanchao Li, Li Xu

**Affiliations:** ^1^ School of Chemistry and Chemical Engineering, Guangdong Pharmaceutical University, Zhongshan, China; ^2^ Guangdong Pharmaceutical University−University of Hong Kong Joint Biomedical Innovation Platform, Zhongshan, China

**Keywords:** N/P co-doping, carbon dots, Fe^3+^ detection, MnO_4_^−^, bacteria imaging

## Abstract

The efficient detection of Fe^3+^ and MnO_4_^−^ in a water environment is very important and challenging due to their harmful effects on the health of humanity and environmental systems. Good biocompatibility, sensitivity, selectivity, and superior photophysical properties were important attributes of carbon dot-based CDs sensors for sensing applications. In this work, we synthesized N, P-*co*-doped carbon dots (N/P CDs) with guanosine 5′-monophosphate (GMP) as a green carbon source, with high fluorescence quantum yield in water (QY, 53.72%). First, the luminescent N/P CDs showed a three-state “on-off-on” fluorescence response upon the sequential addition of Fe^3+^ and F^−^, with a low detection limit of 12 nM for Fe^3+^ and 8.5 nM for F^−^, respectively. Second, the N/P CDs also exhibited desirable selectivity and sensitivity for toxic MnO_4_^−^ detection with the limit of detection of 18.2 nM, through a turn-off mechanism. Moreover, the luminescent N/P CDs successfully monitored the aforementioned ions in environmental water samples and in *Escherichia coli*.

## Introduction

Fe^3+^, F^−^, and MnO_4_^−^ are some well-known important ions. Fe^3+^ is the key and indispensable trace element and plays great roles in many biological processes (D'Autreaux et al., 2005; Hentze et al., 2010; Wu et al., 2011). Both iron shortage and excess will cause some serious functional disorders (Piga et al., 2009; Hyman and Franz, 2012; Torti and Torti, 2013). At the same time, excess Fe^3+^ also will lead to environmental pollution (Zhao et al., 2010). F^−^ is a common additive used in various industries, such as drinking water, pharmaceutical agents, and toothpaste (Kainth et al., 2018). Furthermore, F^−^ belongs to an essential substance and plays a vital role in the human body and many biological processes (Jeong et al., 2018). However, inadequate F^−^ intake is harmful to plants and aquatic organisms. Skeletal and dental fluorosis, kidney and gastric disorders, and DNA damage also have been related to the abnormal distribution of the F^−^ ions (Cametti and Rissanen, 2013; Zheng et al., 2014; Mohapatra et al., 2015). On the other hand, MnO_4_^−^, which exists as the important oxidant in the laboratory and industry, is a class of potent carcinogenic and mutagenic anion that can cause genetic defects, skin allergies and ulcers, and various types of cancers (Thompson et al., 2014; Ding et al., 2016; Liu et al., 2019). Therefore, developing a new effective technology with high sensitivity and rapid response time for detecting hazardous ions is extremely needed in the fields of biological and environmental safety.

Carbon dots (CDs), a new and fascinating nanomaterial, have received considerable concern, owing to their distinctive characteristics, including easy synthesis and functionalization, low cost, good water dispersibility, superior photostability, remarkable biocompatibility, and negligible toxicity (Cao et al., 2007; Baker and Baker, 2010; Wang and Hu, 2014; Lim et al., 2015). Consequently, CDs revealed different applications in drug delivery, bioimaging, optoelectronic devices, and sensing(Zhu et al., 2013; Gong X. et al., 2016; Cailotto et al., 2018; Wu et al., 2019; Su et al., 2020). Until now, a variety of precursors and synthetic methods have been reported. However, the most reported CDs had relatively low QYs, which severely limited their potential applications in living systems. A superior pathway to improve the QYs of CDs was to introduce heteroatoms into CDs (Xu et al., 2016). Considering that heteroatom doping could tailor the surface defects, alter the electronic properties, provide more active moieties, and tune the optical features, they have been constructed as various chemical sensors with fascinating performances in bioimaging and biosensing (Chatzimitakos et al., 2018; Jin et al., 2021; Wang et al., 2021; Fu et al., 2022). Nitrogen (N) and phosphorus (P), which are the most frequently used doping atoms with an atomic size similar to that of carbon and strong binding ability, can affect the optical performances or functions of CDs. Some bright and color-tunable N/P CD-based fluorescent probes have been reported to exhibit highly detectable ability for metal ions and organic molecules by employing various nucleotides as precursors (Gong Y. et al., 2016; Shangguan et al., 2017; Li et al., 2018; Wang et al., 2021). For example, the N/P CDs were constructed to detect Fe^3+^ in biological samples using adenosine−5′−triphosphate (ATP) as a precursor (Shangguan et al., 2017). Li et al. (2018) achieved a picric acid (PA) biosensor with a 30-nM detection limit from adenosine monophosphate (AMP)-derived N/P CDs . However, to the best of our knowledge, there were limited examples of N/P CDs with high QYs prepared from these cheap and sustainable raw materials, and their unique photoluminescence properties were almost completely unknown. Therefore, developing new highly photoluminescent CDs for the luminescent detection by a simple synthesis technique using cheap and green precursors was still highly desired.

In the present work, we developed N/P CDs from GMP by using the hydrothermal technology. The as-prepared N/P CDs had strong blue emission, superior water solubility, high fluorescence QY, and negligible cytotoxicity. The N/P CDs exhibited a high selective and sensitive quenching response toward Fe^3+^ and MnO_4_^−^ in aqueous solutions, and the detection mechanism was also explored. Furthermore, the obtained N/P CDs can be utilized in imaging and detecting the aforementioned ions in bacteria, thus attributing to the good permeability and low cytotoxicity.

## Experiment

### Synthesis of N/P CDs

We prepared the N/P CDs *via* a simple hydrothermal reaction based on a previously reported method (Shangguan et al., 2016). Details on the synthesis of the N/P CDs are provided in the Supporting Information.

### Detection of Selective Ions

Different amounts of analytes (Fe^3+^ and MnO_4_^−^) were gradually added into 10 *μ*g·mL^−1^ N/P CDs, and the fluorescence signals were monitored after incubation for 3 min. In order to further verify the recovery effect of F^−^, Fe^3+^ (40 *μ*M) was added to 10 *μ*g·mL^−1^ N/P CDs, and finally, various concentrations of F^−^ (0–30 *μ*M) were added. To study the selectivity of the N/P CDs for analytes, we introduced other relevant substances into the N/P CD solution and recorded the fluorescence spectrum. The fluorescence emission spectra of each sample were excited at 320 nm and emitted at 392 nm.

### Bacteria Imaging

*Escherichia coli* ATCC 25922 (E. coli) was transferred to Luria-Bertani (LB) broth and incubated in a shaker (200 rpm) at 37°C overnight. Finally, the bacteria optical density at 600 nm (OD 600) was adjusted to reach 1.0.

For the detection of selective ions in bacteria, *E. coli* were cultured with 50 *μ*g mL^−1^ N/P CDs for 4 h. Next, the N/P CD-stained *E. coli* were further treated with FeCl_3_ and KMnO_4_ (200 *μ*M each) solutions for another 2 h at 37°C, respectively. Images of the bacteria were immediately observed by fluorescence microscopy after washing three times with PBS.

## Results and Discussion

### Synthesis and Characterization

**SCHEME 1:**
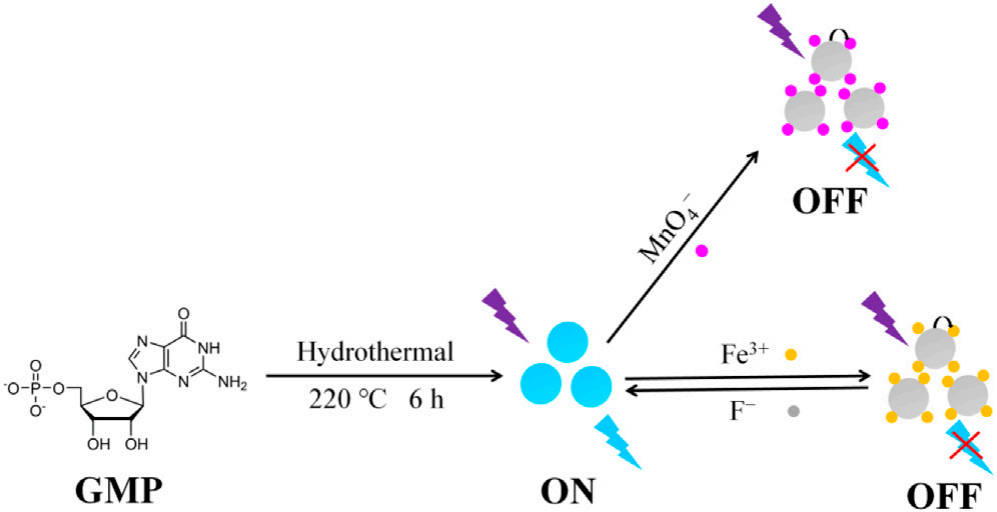
Preparation of the highly fluorescent N/P CDs and their application in Fe^3+^ and MnO_4_^−^ detection.

Blue fluorescent N/P CDs were obtained through hydrothermal treatment using GMP as the only ingredient (Scheme 1). Because of the excellent solubility of GMP, the synthesis process can be carried out in water without the use of any harmful organic reagents, which was well consistent with green chemistry principles. The hydrothermal synthesis conditions were investigated to obtain the optimal optical properties of N/P CDs through a series of experiments (Supplementary Table S1). The highest QY of N/P CDs (53.72%), which was obtained under the optimized hydrothermal condition at 220°C for 6 h, was much higher than other reported CDs (Qu et al., 2012; Shi et al., 2016; Shangguan et al., 2017; Li et al., 2018; Khan et al., 2021). N, P co-doped effect may be the reason for the high QY of N/P CDs, which can modulate the electronic and chemical behaviors of the CDs. Thus, N/P CDs prepared from GMP had great potential as a promising fluorescent nanoprobe due to their high QY.

**FIGURE 1:**
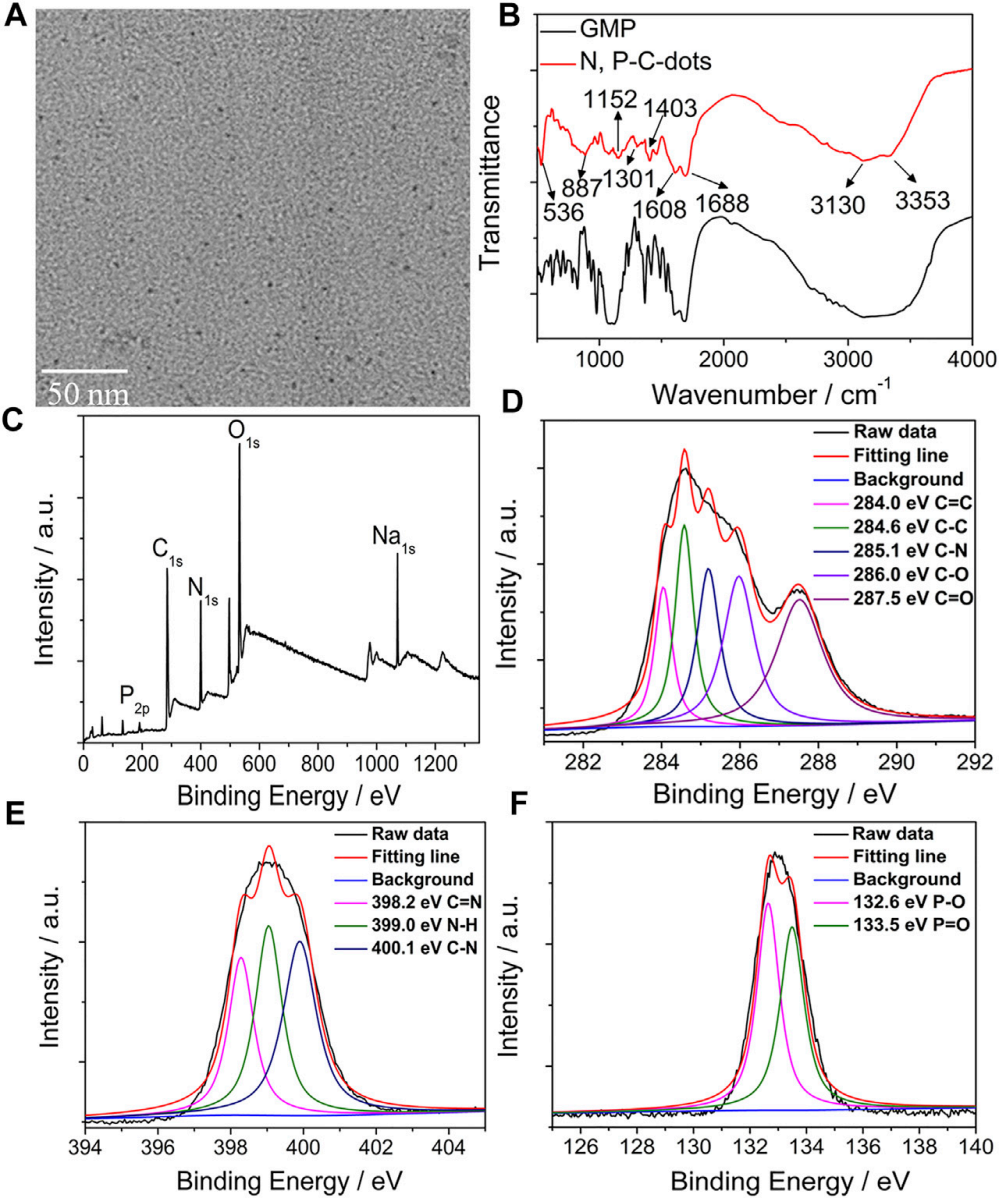
TEM images **(A)**, FT−IR spectrum **(B)**, and XPS spectrum **(C)** and high-resolution C 1s **(D)**, N 1s **(E)**, and P 2p **(F)** XPS spectrum of the N/P CDs.

The microstructure of the N/P CDs was investigated through TEM (Figure 1A), and the N/P CDs presented well dispersed and spherical structure with an average size of ∼3.0 nm (Supplementary Figure S1). The XRD pattern (Supplementary Figure S2) revealed a wide diffraction peak at about 23.4°, indicating a disordered graphite-like structure (Tang et al., 2012). FT-IR spectra confirmed the surface chemistry of the N/P CDs. As illustrated in Figure 1B, the FT-IR spectra of GMP and N/P CDs were performed. Broad absorption peaks at 3,353 and 3,130 cm^−1^ confirmed the stretching vibrations of O−H and N−H bonds present on the N/P CD surface. The peak at 1,688 cm^−1^ indicated C=C and C=O groups. The peaks at 1,608 cm^−1^ and 1,403 cm^−1^ contributed to the bending vibrations of N-H and C-N, respectively (Ananthanarayanan et al., 2015). Furthermore, the stretching vibrations of P−O and P=O appeared at 887, 1,152, and 1,301 cm^−1^, suggesting that the phosphorus element was perfectly adulterated into the C-dots (Du et al., 2014; Shangguan et al., 2017). XPS was performed to analyze the surface states of the N/P CDs. The XPS full survey spectrum (Figure 1C) clearly exhibited that the N/P CDs contained 46.98% carbon (C), 17.06% nitrogen (N), 24.85% oxygen (O), and 3.53% phosphorus (P), and their corresponding peaks were located at 286.1, 399.1, 531.1, and 133.1 eV, respectively. The high-resolution C 1s spectra revealed five major deconvoluted peaks at 284.0 eV (C=C), 284.6 eV (C−C), 285.1 eV (C−N), 286.0 eV (C−O), and 287.5 eV (C=O) (Figure 1D) (Gong X. et al., 2016; Huang et al., 2018). In the deconvoluted N 1s spectrum, three distinct peaks at 398.2, 399.0, and 400.1 eV indicated C=N−H, N−H, and C−N moieties, respectively (Figure 1E) (Huang et al., 2018). Additionally, the high-resolution P 2p spectrum showed two peaks at 132.6 and 133.5 eV, assigned to P−O and P=O, respectively (Figure 1F) (Shangguan et al., 2017). The results of FTIR and XPS spectra suggested that N/P CDs were perfectly adulterated with N and P atoms, and their surfaces contained related functional groups. In addition, the ζ−potential of the N/P CDs was −25.5 mV, suggesting that the surface of the N/P CDs contained the negative functional groups.

### Fluorescence Properties

**FIGURE 2:**
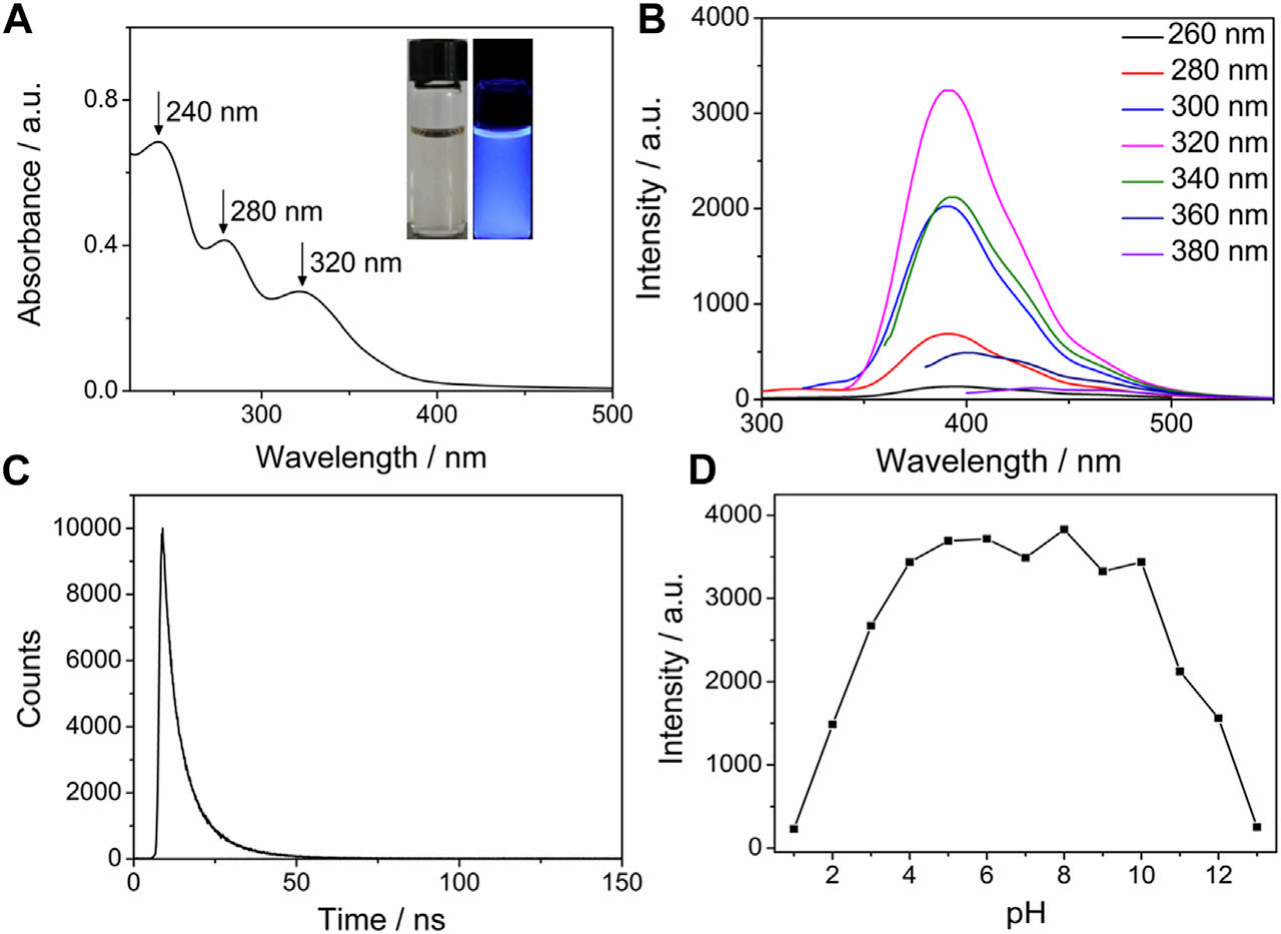
**(A)** Absorption spectrum of N/P CDs (10 *μ*g·mL^−1^). (Insert: N/P CDs solution under daylight (left) and UV light (right)). **(B)** Excitation-dependent emission spectra of N/P CDs (10 *μ*g·mL^−1^). **(C)** Fluorescence decay curve of N/P CDs (10 *μ*g·mL^−1^) under 320 nm excitation. **(D)** Effect of pH on the fluorescence intensities of N/P CDs (10 *μ*g·mL^−1^).

First, we investigated the optical characteristics of the obtained N/P CDs by optical spectroscopy. The UV/vis spectra of N/P CDs contained three dominant absorption bands at 240 nm, 280, and 320 nm, ascribing to π *−*π^∗^ transition of C=C, C=N or N=P bonds, and n*−*π^∗^ transition of C=O bonds, respectively (Figure 2A) (Dong et al., 2013). The N/P CD solution was light brown, transparent, and clear under normal light, while it emitted a bright blue color under a 365 nm UV lamp (inserted in Figure 2A). Under 320 nm excitation, the N/P CDs showed a maximum signal at 392 nm. In addition, by varying excitation wavelength from 260 to 380 nm, the N/P CDs showed a typical excitation-dependent fluorescence feature (Figure 2B). The aforementioned result could be attributed to the impact of the surface states of CDs (Zhou et al., 2019). The QY of N/P CDs excited by 320 nm was as high as 53.72%, which was higher than other reported N/P CDs (Zheng et al., 2013; Gong Y. et al., 2016; Khan et al., 2021; Nandi et al., 2021). The fluorescence lifetime of N/P CDs was fitted by a multi-exponential function, giving two decay times, τ_1_ = 3.93 ns (40.1%) and τ_2_ = 10.55 ns (59.9%), with an average lifetime of 7.89 ns (Figure 2C).

Next, we evaluated the optical stability of the N/P CDs under various conditions, such as pH, salt medium, temperature, and UV-light treatment. The fluorescent signals of N/P CDs at different pH values were investigated, and the results are shown in Figure 2D. At lower and higher pH, N/P CDs showed low fluorescence intensity. However, the fluorescent intensity was maintained constant at a pH range of 4–10. This result may be caused by the protonation−deprotonation of the phosphoric acid group and the amino group on the surface of the N/P CDs (Zhang et al., 2017). Supplementary Figure S3 showed that the N/P CDs remained stable even in a high ionic strength (600 mM NaCl), which was attributed to no ionization of surface functional groups of the N/P CDs (Zhang et al., 2014). At the same time, the fluorescence intensity remained unchanged at the temperatures between 25 and 80°C and continuous illumination for 2 h (Supplementary Figure S4 and Supplementary Figure S5). The excellent thermal stability of the N/P CDs was attributed to the synergic effect of stable composition on the surface of N, P-doped CDs. The oxygen- and nitrogen-rich groups can effectively inhibit the aggregation at a higher temperature and protect the N/P CDs from degradation induced by thermal oxidation (Khan et al., 2018). In addition, the photobleaching-resistant property was possibly ascribed to the electrostatic repulsions between the negatively charged nanoparticles (Huang et al., 2013). The prepared N/P CDs possessed outstanding fluorescence properties and abundant functional groups, which inspired us to investigate their sensing performance.

### Selective Sensing of Fe^3+^ and F^−^

**FIGURE 3:**
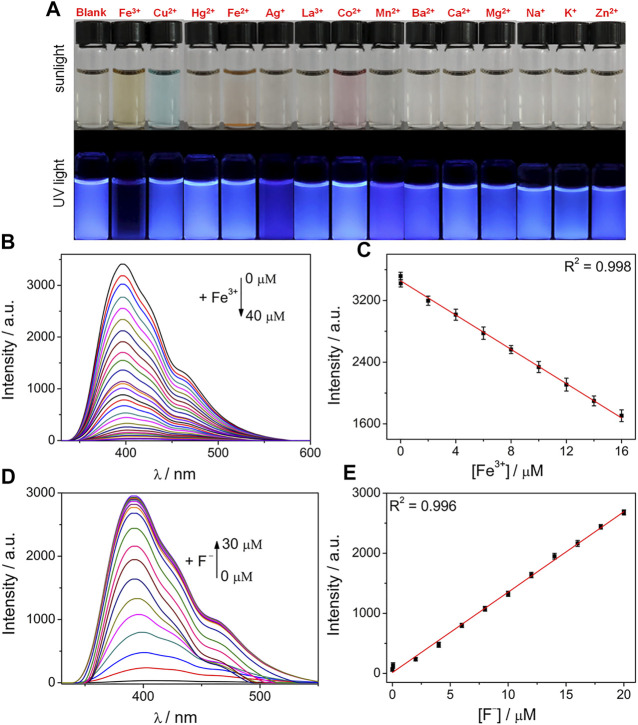
**(A)** Photograph of N/P CDs (10 *μ*g·mL^−1^) mixed with various metal ions (40 *μ*M each) under sunlight and UV light. **(B)** Fluorescence response of N/P CDs (10 *μ*g·mL^−1^) upon addition of various concentrations of Fe^3+^ (0–40 *μ*M). **(C)** Initial linear responses of the fluorescence intensity to low Fe^3+^ concentration (0.01–16 *μ*M). **(D)** Fluorescence spectra of N/P CDs (10 *μ*g·mL^−1^) in the presence of Fe^3+^ (40 *μ*M) and various F^−^ concentrations (0–30 *μ*M). **(E)** Linear relationship of the fluorescence intensity versus F^−^ concentration (0–20 *μ*M).

The N/P CDs were utilized as a promising fluorescence sensor because of their excellent fluorescence properties and water stability. First, we studied the fluorescence response of the prepared CDs toward various metal ions. As presented in Figure 3A, pure N/P CD solution emitted strong blue luminescence, and the fluorescence of the N/P CDs varied from blue to colorless by adding Fe^3+^ ions under UV light. However, the addition of other tested metal ions including Cu^2+^, Hg^2+^, Fe^2+^, Ag^+^, La^3+^, Co^2+^, Mn^2+^, Ba^2+^, Ca^2+^, Mg^2+^, Na^+^, K^+^, and Zn^2+^ showed very little effect on the N/P CD fluorescence. These initial results indicated that N/P CDs can specifically detect Fe^3+^ compared to other tested metal ions. We subsequently evaluated the sensitivity of the prepared N/P CDs toward Fe^3+^ ions by the fluorescence titration experiments. Figure 3B showed a gradual decrease in the fluorescence of N/P CDs with increasing Fe^3+^ concentration and showed 98% fluorescence quenching with 40 *μ*M Fe^3+^. Figure 3C showed a good linear correlation (*R* ^2^ = 0.998) between the intensity at 392 nm and Fe^3+^ concentration (0.01–16 *μ*M). The limit of detection (LOD) was calculated as 12 nM Fe^3+^ based on the formula, LOD = 3σ/slope, where σ was the standard deviation of blank samples. The LOD was well below the LODs of the previously reported CDs (Supplementary Table S2). The N/P CDs/Fe^3+^ system could be further applied for F^−^ determination. The fluorescence signal at 392 nm recovered progressively with increasing F^−^ concentration (Figure 3D). The recovered fluorescent intensity had a good linear correlation (*R* ^2^ = 0.996) with F^−^ concentration (0–20 *μ*M) (Figure 3E). The LOD of F^−^ was calculated as 8.5 nM (3σ/slope).

To evaluate the specificity of N/P CDs in testing Fe^3+^, a series of anti-jamming experiments were carried out (Supplementary Figure S6A). Most metal ions showed neglected influence on Fe^3+^ detection, indicating that N/P CDs had excellent selectivity for Fe^3+^. On the other hand, the anti-interference sensing ability of the N/P CDs/Fe^3+^ system toward F^−^ was further ascertained by the competing experiments. Only F^−^ recovered the fluorescence of the N/P CDs/Fe^3+^ system; other anions did not cause obvious fluorescence enhancements (Supplementary Figure S6B).

In order to further confirm that the system we developed had potential biological applications, the fluorescence response of N/P CDs toward Fe^3+^ and F^−^ was explored in HEPES buffer at different pH ranges. N/P CDs can maintain stable fluorescence from pH 4–8 (Supplementary Figure S7), indicating their outstanding photostability in a wide pH range. A notable fluorescence quenching was observed after adding Fe^3+^ with pH from 1 to 12, suggesting that N/P CDs were effectively quenched by Fe^3+^ from pH 1–12. In addition, a stable fluorescence-recovered phenomenon of N/P CDs/Fe^3+^ system to F^−^ was observed at pH 4–10. Thus, N/P CDs can sequentially detect Fe^3+^ and F^−^ at biological pH values.

Time-dependent fluorescence variation of N/P CDs to Fe^3+^/F^−^ sequential detection was investigated (Supplementary Figure S8). N/P CDs maintained stable fluorescence under aqueous media, indicating good water solubility and stability abilities. However, with the addition of Fe^3+^, the fluorescence of N/P CDs was rapidly quenched within 30 s and then remained stable thereafter (Supplementary Figure S8A). Subsequent addition of F^−^ to the CDs/Fe^3+^ system resulted in fluorescence recovery within 30 s, which indicated that Fe^3+^ can be released from N/P CD surfaces (Supplementary Figure S8B). The aforementioned results demonstrated that N/P CDs could be employed for the real-time Fe^3+^ and F^−^ determination.

Reusability was an important characteristic of a probe used for practical detection. A switchable change in the fluorescence signal at 392 nm could be repeated, and no obvious signal loss was detected by the alternate introduction of Fe^3+^ and F^−^ (Supplementary Figure S9), indicating that N/P CDs can be reused for Fe^3+^ and F^−^ detection.

**FIGURE 4:**
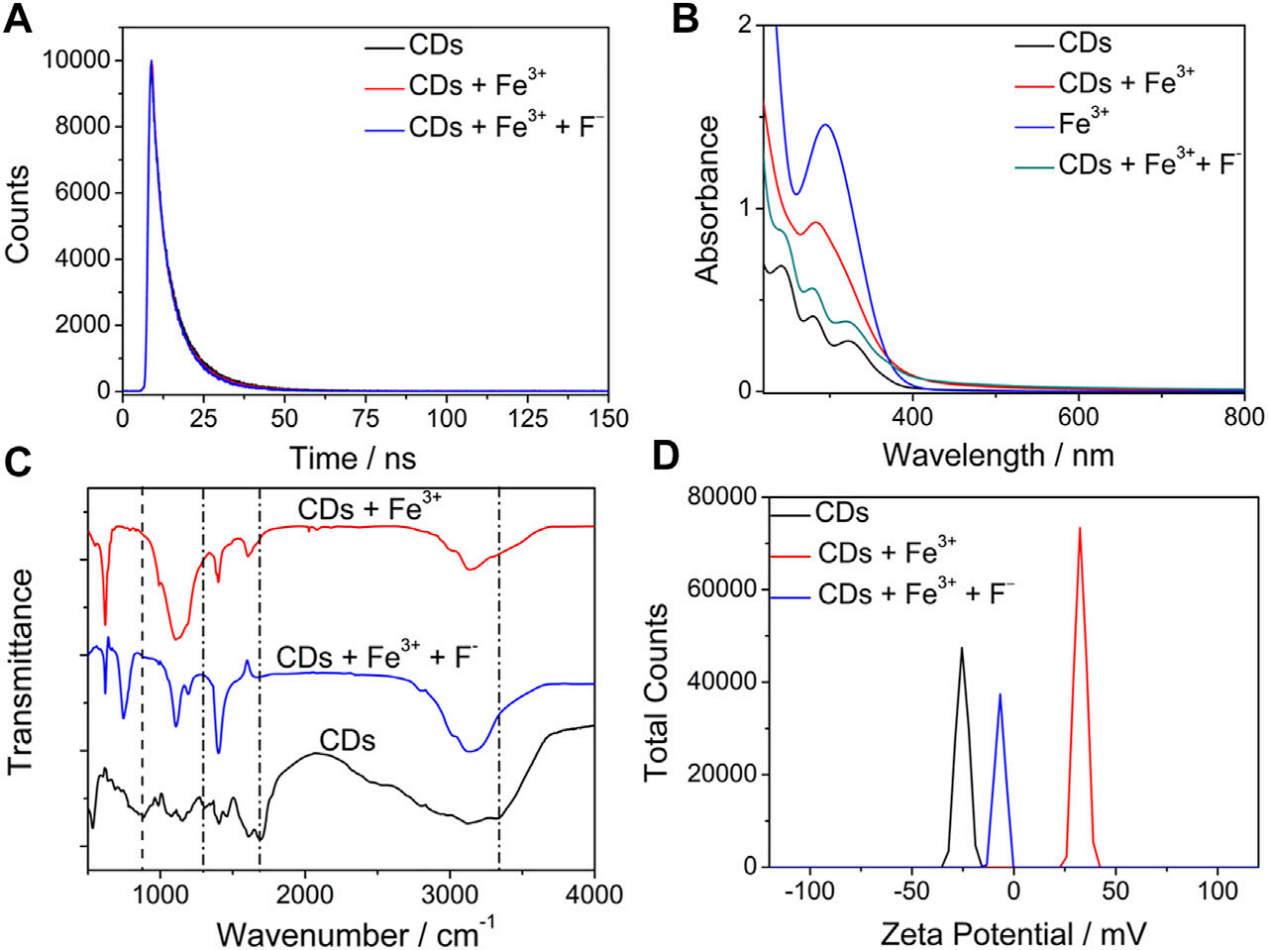
**(A)** Fluorescence decay curves of the N/P CDs (10 *μ*g·mL^−1^) with and without Fe^3+^ (40 *μ*M) and F^−^ (30 *μ*M). **(B)** UV−vis spectra of Fe^3+^, N/P CDs, and N/P CDs in the absence and presence of Fe^3+^ (40 *μ*M) and F^−^ (30 *μ*M). **(C)** FTIR spectra and **(D)** ζ potential of the N/P CDs (10 *μ*g·mL^−1^) with and without Fe^3+^ (40 *μ*M) and F^−^ (30 *μ*M).

The interaction between Fe^3+^ and the multifunctional groups (amino, phosphoric acid, and carboxyl groups) of N/P CDs could modulate the fluorescence properties. Fe^3+^ quenched the fluorescence of N/P CDs because of the strong complexation interaction between them (Mohammed and Omer, 2020). The average fluorescence lifetime of N/P CDs was 7.89 ns However, with Fe^3+^ and F^−^, the average lifetimes of N/P CDs were 7.85 [τ_1_ = 4.48 ns (40.7%) and τ_2_ = 10.16 ns (59.3%)] and 7.87 [τ_1_ = 5.16 ns (35.3%) and τ_2_ = 9.35 ns (64.7%)] ns (Figure 4A), respectively, suggesting Fe^3+^−induced fluorescence quenching was probably a static quenching process (Lu et al., 2017). To further clarify this deduction, UV/vis absorption spectra, FT-IR, and zeta-potential analysis were implemented. Absorption spectra of N/P CDs were notably affected and changed after the addition of Fe^3+^ (Figure 4B), which was attributable to the strong chemical interaction between Fe^3+^ and N/P CDs followed by stable metal complex formation (Rajendran et al., 2021). Absorption peaks of N/P CDs shifted to their former position again with the addition of F^−^. Comparing the N/P CDs before and after treating with Fe^3+^, the characteristic vibration peaks of C=O, P−O, and P=O groups (1,688, 887, and 1,301 cm^−1^) demonstrated obvious changes (Figure 4C) (Shangguan et al., 2017). Meanwhile, the zeta potential increased from −25.5 to +32.4 mV when Fe^3+^ was present and then reversed back to −6.7 mV for the N/P CDs/Fe^3+^ system after treating with F^−^ (Figure 4D), which proved that Fe^3+^ was indeed selectively complexed with the negatively charged groups of the CDs.

### Selective Sensing of MnO_4_^−^

**FIGURE 5:**
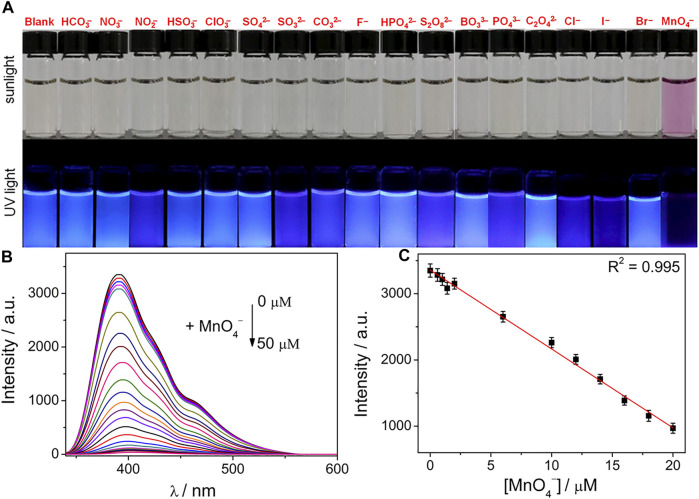
**(A)** Photographs of N/P CD solution in the presence of various anions (50 *μ*M) under sunlight and UV light (365 nm). **(B)** Fluorescence response of N/P CDs (10 *μ*g·mL^−1^) upon addition of various concentrations of MnO_4_^−^ (0–50 *μ*M) in HEPES buffer (10 mM, pH 7.4). **(C)** Linear relationship of the fluorescence intensity against MnO_4_^−^ concentrations.

Simultaneously, various anions were used to demonstrate the anion-sensing abilities of the N/P CDs based on similar research methods for metal cations. In order to evaluate the sensing behaviors of the N/P CDs toward anions, we separately added 19 anionic salt aqueous solutions (50 *μ*M) to the aqueous suspension of N/P CDs (10 *μ*g·mL^−1^). The fluorescence of the N/P CDs changed from blue to colorless in the case of MnO_4_^−^ treatment under UV light excitation (365 nm) (Figure 5A). However, adding other tested anions did not induce conspicuous fluorescence changes in the N/P CD solution.

The sensitivity of the N/P CDs toward MnO_4_^−^ in water was explored by performing the concentration−dependent titration experiments. Upon introducing various concentrations of the MnO_4_^−^ (0–50 *μ*M) into the N/P CD solution (10 *μ*g·mL^−1^), the fluorescence signals of the N/P CDs gradually decreased and showed a complete quenching response by MnO_4_^−^ (Figure 5B) at the maximum concentration. Furthermore, Figure 5C showed a good linear relationship of the fluorescence values at 392 nm versus the concentrations of MnO_4_^−^ (0–20 *μ*M). The LOD of MnO_4_^−^ was 18.2 nM. The detection performance of the N/P CDs for MnO_4_^−^ in this work was better than that of the previously reported literature presented in Supplementary Table S3.

The competition tests should be conducted to confirm the muscular anti-interference ability of N/P CDs toward MnO_4_^−^ in the presence of other 17 inorganic anions (Supplementary Figure S10A). The results indicated that other interfering anions did not induce significant changes in fluorescence, suggesting that MnO_4_^−^ anions can be selectively distinguished by N/P CDs in the coexistence of other inorganic anions.

Time-dependent fluorescence fluctuation to detect MnO_4_^−^ with N/P CDs was explored. When mixing various concentrations of MnO_4_^−^ with N/P CD (10 *μ*g·mL^−1^) suspension, the fluorescence signal progressively decreased (Supplementary Figure S10B). In addition, the N/P CDs showed a significant quenching effect on MnO_4_^−^ at various pH ranges (1–13) (Supplementary Figure S10C), which can be applied to detect and image MnO_4_^−^ in biological systems.

To clarify the detection mechanism, we recorded UV−vis spectra of the MnO_4_^−^ in water (Supplementary Figure S11A). The excitation spectra of N/P CDs (260–380 nm) displayed broad overlaps with UV−vis spectra of MnO_4_^−^ (270–600 nm), suggesting that the internal filtration effect (IFE) was responsible for fluorescence quenching (Li et al., 2018; Ji et al., 2020). Additionally, the emission spectra of N/P CDs overlapped with the UV−vis spectrum of MnO_4_^−^, indicating that resonance energy transfer (RET) was possibly produced from N/P CDs to analytes (Li et al., 2018). To further confirm whether RET happened during the MnO_4_^−^ sensing process, the fluorescence lifetimes of N/P CDs were measured with or without MnO_4_^−^. Lifetimes were as follows: 7.89 ns (before the addition of MnO_4_^−^) and 7.87 ns (after the addition of MnO_4_^−^; Supplementary Figure S11B). The lifetimes had almost no change in the presence of MnO_4_^−^, which indicated that the static quenching effect (SQE) was formed between N/P CDs and MnO_4_^−^. In the RET process, target molecules can significantly change the fluorescence lifetime of the fluorophore (Joseph and Anappara, 2016; Liu et al., 2016; Sun et al., 2016; Li et al., 2018). Thus, RET was not the main reason to induce the fluorescence quenching. In conclusion, IFE and SQE played a main role in inducing the fluorescence quenching process.

### Detection of Multiple Analytes in Real Water Samples

To demonstrate the potential analytical application in a real sample, quantitative analytes’ (Fe^3+^, F^−^, and MnO_4_^−^) detection tests were carried out in lake water by the standard spiking method. The results are listed in Supplementary Table S4, and the recoveries changed between 97.6 and 102.9% with relative standard deviations (RSDs) lower than 1.1%. Therefore, N/P CDs had excellent potential for these analytes’ detection in actual water samples.

### Detection of Multiple Analytes in Bacteria

**FIGURE 6:**
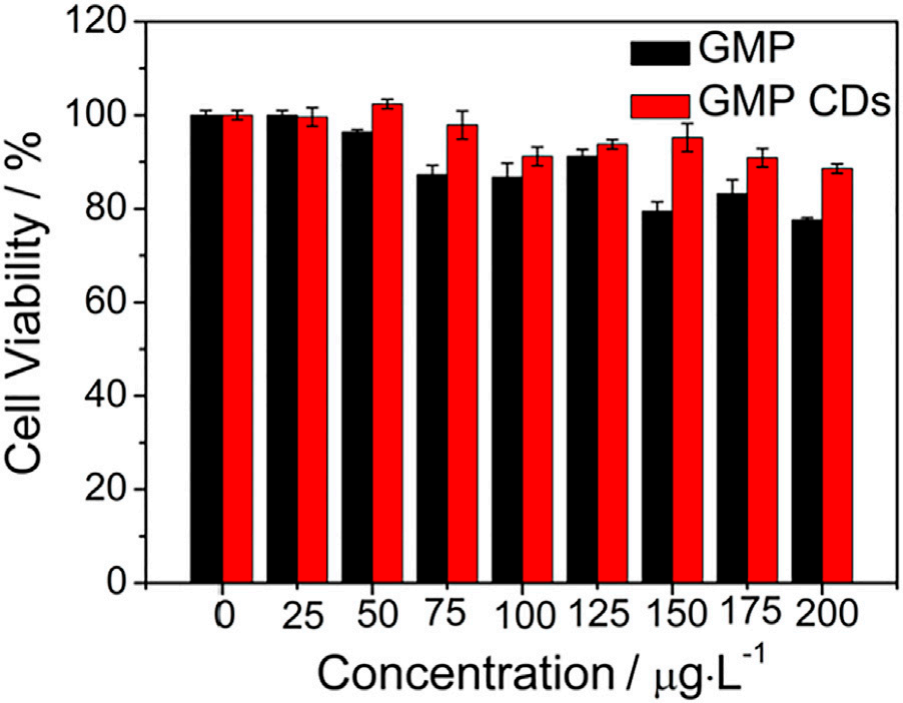
Viability of HeLa cells treated with N/P CDs at various concentrations (0–200 *μ*g·mL^−1^) for 24 h.

**FIGURE 7:**
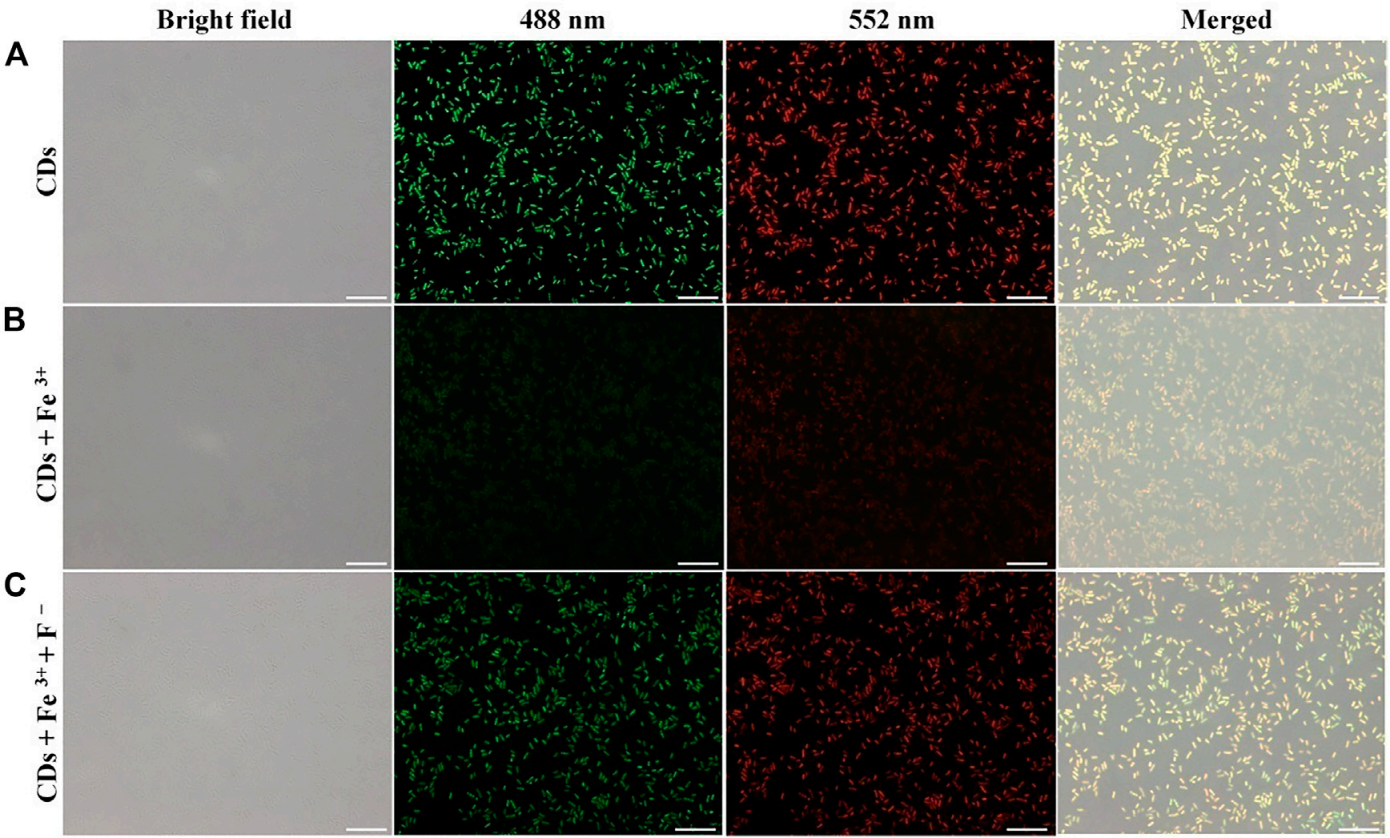
Bright-field, fluorescence, and the overlaid images of *E. coli*. *E. coli* was incubated with N/P CDs **(A)**; *E. coli* was incubated with N/P CDs and then treated with Fe^3+^ **(B)**; *E. coli* was incubated with N/P CDs, Fe^3+^, and then treated with F^−^ **(C)**. Images were captured with 488 (green) and 552 nm laser (red). The scale bars represented 10 *μ*m.

To study the potential biomedical applications of the N/P CDs, the cytotoxicity assay was first examined using HeLa cells by the traditional MTT method. Approximately 90% of cells still survived even with 200 *μ*g·mL^−1^ N/P CDs for 24 h (Figure 6), suggesting that N/P CDs were nontoxic in nature and displayed good biocompatibility. In order to investigate the potential biological imaging functions of N/P CDs, fluorescence imaging was performed in *E. coli* to monitor Fe^3+^ and F^−^. As displayed in Figure 7, E. coli stained by 50 *μ*g·mL^−1^ N/P CDs emitted bright green and red emissions when stimulated by 488 and 552 nm lasers, respectively, suggesting that N/P CDs were internalized by *E. coli*. After treatment with 200 *μ*M Fe^3+^ for 30 min, the fluorescence signals of *E. coli* with green and red emissions disappeared. Subsequently, the fluorescence recovered when 150 *μ*M of F^−^ was treated with the aforementioned bacteria, and an evident “on−off−on” fluorescence response appeared. Subsequently, the N/P CDs also were employed to detect MnO_4_^−^ in *E. coli* by fluorescence microscopy. As shown in Supplementary Figure S12, no emission was observed for MnO_4_^−^ (200 *μ*M)-treated *E. coli* under 488, and 552 nm laser excitation, respectively. But without MnO_4_^−^, a bright green and red emission was observed for only N/P CDs (50 *μ*g·mL^−1^) treated with *E. coli*. These results suggested that the N/P CDs could be applied in the visual detection of Fe^3+^, F^−^, and MnO_4_^−^ in bacteria and possessed great promise in bioimaging and biosensing applications.

## Conclusions

In summary, we synthesized bright blue fluorescent N/P CDs from GMP. The obtained N/P CDs possessed excellent stability under various situations (UV irradiation, pH treatment, and temperature), low toxicity, and high quantum yields (53.72%). The N/P CDs were endowed with high sensitivity and were selective toward Fe^3+^ and MnO_4_^−^ with respective detection limits of 12 and 18.2 nM. Furthermore, we further applied the proposed fluorescent sensor to detect the aforementioned ions in real samples with a satisfactory outcome. Finally, the N/P CDs were introduced for sensing the aforementioned ions in bacteria. This work fabricated an excellent fluorescent sensor for quantitatively monitoring Fe^3+^ and MnO_4_^−^ and also enriched the design of CD-based nanosensors.

## Data Availability

The original contributions presented in the study are included in the article/Supplementary Material; further inquiries can be directed to the corresponding authors.

## References

[B1] Ananthanarayanan A. Wang Y. Routh P. Sk M. A. Than A. Lin M. (2015). Nitrogen and Phosphorus Co-doped Graphene Quantum Dots: Synthesis from Adenosine Triphosphate, Optical Properties, and Cellular Imaging. Nanoscale 7, 8159–8165. 10.1039/c5nr01519g 25875153

[B2] Baker S. N. Baker G. A. (2010). Luminescent Carbon Nanodots: Emergent Nanolights. Angew. Chem. Int. Ed. 49, 6726–6744. 10.1002/anie.200906623 20687055

[B3] Cailotto S. Mazzaro R. Enrichi F. Vomiero A. Selva M. Cattaruzza E. (2018). Design of Carbon Dots for Metal-free Photoredox Catalysis. ACS Appl. Mat. Interfaces 10, 40560–40567. 10.1021/acsami.8b14188 30370767

[B4] Cametti M. Rissanen K. (2013). Highlights on Contemporary Recognition and Sensing of Fluoride Anion in Solution and in the Solid State. Chem. Soc. Rev. 42, 2016–2038. 10.1039/c2cs35439j 23188119

[B5] Cao L. Wang X. Meziani M. J. Lu F. Wang H. Luo P. G. (2007). Carbon Dots for Multiphoton Bioimaging. J. Am. Chem. Soc. 129, 11318–11319. 10.1021/ja073527l 17722926 PMC2691414

[B6] Chatzimitakos T. Kasouni A. Sygellou L. Leonardos I. Troganis A. Stalikas C. (2018). Human Fingernails as an Intriguing Precursor for the Synthesis of Nitrogen and Sulfur-Doped Carbon Dots with Strong Fluorescent Properties: Analytical and Bioimaging Applications. Sensors Actuators B Chem. 267, 494–501. 10.1016/j.snb.2018.04.059

[B7] D'Autréaux B. Tucker N. P. Dixon R. Spiro S. (2005). A Non-haem Iron Centre in the Transcription Factor NorR Senses Nitric Oxide. Nature 437, 769–772. 10.1038/nature03953 16193057

[B8] Ding B. Liu S. X. Cheng Y. Guo C. Wu X. X. Guo J. H. (2016). Heterometallic Alkaline Earth-Lanthanide BaII-LaIII Microporous Metal-Organic Framework as Bifunctional Luminescent Probes of Al3+ and MnO4-. Inorg. Chem. 55, 4391–4402. 10.1021/acs.inorgchem.6b00111 27088966

[B9] Dong Y. Pang H. Yang H. B. Guo C. Shao J. Chi Y. (2013). Carbon-Based Dots Co-doped with Nitrogen and Sulfur for High Quantum Yield and Excitation-independent Emission. Angew. Chem. Int. Ed. 52 (30), 7800–7804. 10.1002/anie.201301114 23761198

[B10] Du F. Y. Jin X. Chen J. H. Hua Y. Cao M. L. Zhang L. R. (2014). Nitrogen-doped Carbon Dots as Multifunctional Fluorescent Probes. J. Nanopart. Res. 16 (11), 1–10. 10.1007/s11051-014-2720-8

[B11] Fu M.-J. Wei N. Pang L.-F. Guo X.-F. Wang H. (2022). Red Emission Nitrogen and Zinc Co-doped Carbon Dots as Fluorescent Sensor for Reversible Detection of Peroxynitrite in Living Cells. Sensors Actuators B Chem. 351, 130939. 10.1016/j.snb.2021.130939

[B12] Gong X. Zhang Q. Gao Y. Shuang S. Choi M. M. F. Dong C. (2016a). Phosphorus and Nitrogen Dual-Doped Hollow Carbon Dot as a Nanocarrier for Doxorubicin Delivery and Biological Imaging. ACS Appl. Mat. Interfaces 8, 11288–11297. 10.1021/acsami.6b01577 27088972

[B13] Gong Y. Yu B. Yang W. Zhang X. (2016b). Phosphorus, and Nitrogen Co-doped Carbon Dots as a Fluorescent Probe for Real-Time Measurement of Reactive Oxygen and Nitrogen Species inside Macrophages. Biosens. Bioelectron. 79, 822–828. 10.1016/j.bios.2016.01.022 26774996

[B14] Hentze M. W. Muckenthaler M. U. Galy B. Camaschella C. (2010). Two to Tango: Regulation of Mammalian Iron Metabolism. Cell. 142, 24–38. 10.1016/j.cell.2010.06.028 20603012

[B15] Huang H. Lv J.-J. Zhou D.-L. Bao N. Xu Y. Wang A.-J. (2013). One-pot Green Synthesis of Nitrogen-Doped Carbon Nanoparticles as Fluorescent Probes for Mercury Ions. RSC Adv. 3, 21691–21696. 10.1039/c3ra43452d

[B16] Huang Q. Li Q. Chen Y. Tong L. Lin X. Zhu J. (2018). High Quantum Yield Nitrogen-Doped Carbon Dots: Green Synthesis and Application as "Off-On" Fluorescent Sensors for the Determination of Fe3+ and Adenosine Triphosphate in Biological Samples. Sensors Actuators B Chem. 276, 82–88. 10.1016/j.snb.2018.08.089

[B17] Hyman L. M. Franz K. J. (2012). Probing Oxidative Stress: Small Molecule Fluorescent Sensors of Metal Ions, Reactive Oxygen Species, and Thiols. Coord. Chem. Rev. 256, 2333–2356. 10.1016/j.ccr.2012.03.009 23440254 PMC3579673

[B18] Jeong S. Kim D. Kim Y.-T. Yoon H.-O. (2018). A Rapid Screening of Fluorine Contents in Soil with a Consideration of Chemical Binding by Wavelength Dispersive X-Ray Fluorescence Spectrometry. Spectrochim. Acta Part B At. Spectrosc. 149, 261–266. 10.1016/j.sab.2018.08.007

[B19] Ji C. Zhou Y. Leblanc R. M. Peng Z. (2020). Recent Developments of Carbon Dots in Biosensing: A Review. ACS Sens. 5, 2724–2741. 10.1021/acssensors.0c01556 32812427

[B20] Jin J.-C. Yu Y. Yan R. Cai S.-L. Zhang X.-Y. Jiang F.-L. (2021). N,S-codoped Carbon Dots with Red Fluorescence and Their Cellular Imaging. ACS Appl. Bio Mat. 4, 4973–4981. 10.1021/acsabm.1c00242 35007045

[B21] Joseph J. Anappara A. A. (2016). Microwave-assisted Hydrothermal Synthesis of UV-Emitting Carbon Dots from Tannic Acid. New J. Chem. 40, 8110–8117. 10.1039/c6nj02107g

[B22] Kainth S. Mehta A. Mishra A. Basu S. (2018). Implementation of a Logic Gate by Chemically Induced Nitrogen and Oxygen Rich C-Dots for the Selective Detection of Fluoride Ions. New J. Chem. 42, 12162–12171. 10.1039/c8nj02041h

[B23] Khan W. U. Qin L. Alam A. Zhou P. Peng Y. Wang Y. (2021). Fluorescent Carbon Dots an Effective Nano-Thermometer *In Vitro* Applications. ACS Appl. Bio Mat. 4, 5796. 10.1021/acsabm.1c00528 35006753

[B24] Khan W. U. Wang D. Wang Y. (2018). Highly Green Emissive Nitrogen-Doped Carbon Dots with Excellent Thermal Stability for Bioimaging and Solid-State LED. Inorg. Chem. 57, 15229–15239. 10.1021/acs.inorgchem.8b02524 30495940

[B25] Li N. Liu S. G. Fan Y. Z. Ju Y. J. Xiao N. Luo H. Q. (2018). Adenosine-derived Doped Carbon Dots: from an Insight into Effect of N/P Co-doping on Emission to Highly Sensitive Picric Acid Sensing. Anal. Chim. Acta 1013, 63–70. 10.1016/j.aca.2018.01.049 29501093

[B26] Lim S. Y. Shen W. Gao Z. (2015). Carbon Quantum Dots and Their Applications. Chem. Soc. Rev. 44, 362–381. 10.1039/c4cs00269e 25316556

[B27] Liu H. Rong J. Shen G. Song Y. Gu W. Liu X. (2019). A Fluorescent Probe for Sequential Sensing of MnO4− and Cr2O72− Ions in Aqueous Medium Based on a UCNS/TMB Nanosystem. Dalton Trans. 48, 4168–4175. 10.1039/c9dt00360f 30869717

[B28] Liu S. G. Luo D. Li N. Zhang W. Lei J. L. Li N. B. (2016). Water-soluble Nonconjugated Polymer Nanoparticles with Strong Fluorescence Emission for Selective and Sensitive Detection of Nitro-Explosive Picric Acid in Aqueous Medium. ACS Appl. Mat. Interfaces 8, 21700–21709. 10.1021/acsami.6b07407 27471907

[B29] Lu W. Gao Y. Jiao Y. Shuang S. Li C. Dong C. (2017). Carbon Nano-Dots as a Fluorescent and Colorimetric Dual-Readout Probe for the Detection of Arginine and Cu2+and its Logic Gate Operation. Nanoscale 9, 11545–11552. 10.1039/c7nr02336g 28770932

[B30] Mohammed L. J. Omer K. M. (2020). Dual Functional Highly Luminescence B, N Co-doped Carbon Nanodots as Nanothermometer and Fe3+/Fe2+ Sensor. Sci. Rep. 10 (1), 3028. 10.1038/s41598-020-59958-5 32080282 PMC7033239

[B31] Mohapatra S. Sahu S. Nayak S. Ghosh S. K. (2015). Design of Fe3O4@SiO2@Carbon Quantum Dot Based Nanostructure for Fluorescence Sensing, Magnetic Separation, and Live Cell Imaging of Fluoride Ion. Langmuir 31, 8111–8120. 10.1021/acs.langmuir.5b01513 26114840

[B32] Nandi N. Gaurav S. Sarkar P. Kumar S. Sahu K. (2021). Multifunctional N-Doped Carbon Dots for Bimodal Detection of Bilirubin and Vitamin B12, Living Cell Imaging, and Fluorescent Ink. ACS Appl. Bio Mat. 4, 5201–5211. 10.1021/acsabm.1c00371 35007002

[B33] Piga A. Longo F. Duca L. Roggero S. Vinciguerra T. Calabrese R. (2009). High Nontransferrin Bound Iron Levels and Heart Disease in Thalassemia Major. Am. J. Hematol. 84 (1), 29–33. 10.1002/ajh.21317 19006228

[B34] Qu S. Wang X. Lu Q. Liu X. Wang L. (2012). A Biocompatible Fluorescent Ink Based on Water-Soluble Luminescent Carbon Nanodots. Angew. Chem. Int. Ed. 51, 12215–12218. 10.1002/anie.201206791 23109224

[B35] Rajendran S. Zichri S. B. Usha Vipinachandran V. Jelinek R. Bhunia S. K. (2021). Triphenylphosphonium‐Derived Bright Green Fluorescent Carbon Dots for Mitochondrial Targeting and Rapid Selective Detection of Tetracycline. ChemNanoMat 7 (5), 545–552. 10.1002/cnma.202100125

[B36] Shangguan J. He D. He X. Wang K. Xu F. Liu J. (2016). Label-free Carbon-Dots-Based Ratiometric Fluorescence pH Nanoprobes for Intracellular pH Sensing. Anal. Chem. 88, 7837. 10.1021/acs.analchem.6b01932 27334762

[B37] Shangguan J. Huang J. He D. He X. Wang K. Ye R. (2017). Highly Fe3+-Selective Fluorescent Nanoprobe Based on Ultrabright N/P Codoped Carbon Dots and its Application in Biological Samples. Anal. Chem. 89, 7477. 10.1021/acs.analchem.7b01053 28628302

[B38] Shi B. Su Y. Zhang L. Huang M. Liu R. Zhao S. (2016). Nitrogen and Phosphorus Co-doped Carbon Nanodots as a Novel Fluorescent Probe for Highly Sensitive Detection of Fe3+ in Human Serum and Living Cells. ACS Appl. Mat. Interfaces 8, 10717–10725. 10.1021/acsami.6b01325 27014959

[B39] Su W. Guo R. Yuan F. Li Y. Li X. Zhang Y. (2020). Red-emissive Carbon Quantum Dots for Nuclear Drug Delivery in Cancer Stem Cells. J. Phys. Chem. Lett. 11, 1357–1363. 10.1021/acs.jpclett.9b03891 32017568

[B40] Sun X. He J. Meng Y. Zhang L. Zhang S. Ma X. (2016). Microwave-assisted Ultrafast and Facile Synthesis of Fluorescent Carbon Nanoparticles from a Single Precursor: Preparation, Characterization and Their Application for the Highly Selective Detection of Explosive Picric Acid. J. Mat. Chem. A 4, 4161–4171. 10.1039/c5ta10027e

[B41] Tang L. Ji R. Cao X. Lin J. Jiang H. Li X. (2012). Deep Ultraviolet Photoluminescence of Water-Soluble Self-Passivated Graphene Quantum Dots. ACS Nano 6 (6), 5102–5110. 10.1021/nn300760g 22559247

[B42] Thompson C. M. Kirman C. R. Proctor D. M. Haws L. C. Suh M. Hays S. M. (2014). A Chronic Oral Reference Dose for Hexavalent Chromium‐induced Intestinal Cancer. J. Appl. Toxicol. 34, 525–536. 10.1002/jat.2907 23943231 PMC4282340

[B43] Torti S. V. Torti F. M. (2013). Iron and Cancer: More Ore to Be Mined. Nat. Rev. Cancer 13 (5), 342–355. 10.1038/nrc3495 23594855 PMC4036554

[B44] Wang X. Yu J. Ji W. Arabi M. Fu L. Li B. (2021). On-Off-On Fluorescent Chemosensors Based on N/P-Codoped Carbon Dots for Detection of Microcystin-LR. ACS Appl. Nano Mat. 4, 6852–6860. 10.1021/acsanm.1c00921

[B45] Wang Y. Hu A. (2014). Carbon Quantum Dots: Synthesis, Properties and Applications. J. Mat. Chem. C 2, 6921–6939. 10.1039/c4tc00988f

[B46] Wu B. Zhu G. Dufresne A. Lin N. (2019). Fluorescent Aerogels Based on Chemical Crosslinking between Nanocellulose and Carbon Dots for Optical Sensor. ACS Appl. Mat. Interfaces 11, 16048–16058. 10.1021/acsami.9b02754 30977364

[B47] Wu J. Liu W. Ge J. Zhang H. Wang P. (2011). New Sensing Mechanisms for Design of Fluorescent Chemosensors Emerging in Recent Years. Chem. Soc. Rev. 40, 3483–3495. 10.1039/c0cs00224k 21445455

[B48] Xu Q. Kuang T. Liu Y. Cai L. Peng X. Sreenivasan Sreeprasad T. (2016). Heteroatom-doped Carbon Dots: Synthesis, Characterization, Properties, Photoluminescence Mechanism and Biological Applications. J. Mat. Chem. B 4, 7204–7219. 10.1039/c6tb02131j 32263722

[B49] Zhang H. Chen Y. Liang M. Xu L. Qi S. Chen H. (2014). Solid-phase Synthesis of Highly Fluorescent Nitrogen-Doped Carbon Dots for Sensitive and Selective Probing Ferric Ions in Living Cells. Anal. Chem. 86, 9846. 10.1021/ac502446m 25211236

[B50] Zhang M. Wang W. Yuan P. Chi C. Zhang J. Zhou N. (2017). Synthesis of Lanthanum Doped Carbon Dots for Detection of Mercury Ion, Multi-Color Imaging of Cells and Tissue, and Bacteriostasis. Chem. Eng. J. 330, 1137–1147. 10.1016/j.cej.2017.07.166

[B51] Zhao Q. Li F. Huang C. (2010). Phosphorescent Chemosensors Based on Heavy-Metal Complexes. Chem. Soc. Rev. 39, 3007. 10.1039/b915340c 20480067

[B52] Zheng X. T. Than A. Ananthanaraya A. Kim D.-H. Chen P. (2013). Graphene Quantum Dots as Universal Fluorophores and Their Use in Revealing Regulated Trafficking of Insulin Receptors in Adipocytes. ACS Nano 7, 6278–6286. 10.1021/nn4023137 23799995

[B53] Zheng X. Zhu W. Liu D. Ai H. Huang Y. Lu Z. (2014). Highly Selective Colorimetric/fluorometric Dual-Channel Fluoride Ion Probe, and its Capability of Differentiating Cancer Cells. ACS Appl. Mat. Interfaces 6, 7996–8000. 10.1021/am501546h 24832790

[B54] Zhou Y. Zahran E. M. Quiroga B. A. Perez J. Mintz K. J. Peng Z. (2019). Size-dependent Photocatalytic Activity of Carbon Dots with Surface-State Determined Photoluminescence. Appl. Catal. B Environ. 248, 157–166. 10.1016/j.apcatb.2019.02.019 PMC7434043 32831482

[B55] Zhu S. Meng Q. Wang L. Zhang J. Song Y. Jin H. (2013). Highly Photoluminescent Carbon Dots for Multicolor Patterning, Sensors, and Bioimaging. Angew. Chem. Int. Ed. 52, 3953–3957. 10.1002/anie.201300519 23450679

