# Supplementary material for: Highly Luminescent Nucleoside-Based N, P-Doped Carbon Dots for Sensitive Detection of Ions and Bioimaging
Source: Front Chem. 2022 Jun 7;10:906806. doi: 10.3389/fchem.2022.906806 (PMC9210210; doi:10.3389/fchem.2022.906806)
Supplement: Supplementary file 1 [file DataSheet1.doc]

Supplementary Information

Highly luminescent nucleoside-based N, P-doped carbon dots for sensitive detection of ions and bioimaging

**Mengru Wang^1^, Mengling Liu^1^, Shuli Nong^1^, Wenzhu Song^1^, Xianpeng Zhang^1^, Shuang Shen^1^, Guohong Jian^1^, Xiangyao Chen^1^, Zhanchao Li^*1^, Li Xu^*1,2^**

*^1^School of Chemistry and Chemical Engineering, Guangdong Pharmaceutical University, Zhongshan, 528458, P. R. China*

*^2^GDPU-HKU Zhongshan Biomedical Innovation Platform, Zhongshan, 528458, P. R. China*

* Corresponding author. Tel: +86-760-88207266; fax: +86-760-88207266

*E-mail address:* [xuli473@163.com (L](mailto:xuli473@163.com (L). Xu); zhanchao8052@gdpu.edu.cn.

**Table of contents**

**1.** Materials

**2.** Apparatus

**3.** Preparation of N/P CDs

**4.** Quantum yield (QY) measurement

**5.** Analysis of analyte in real samples

**6.** Cytotoxicity assays

**Figure S1.** Size distribution of N/P CDs.

**Figure S2.** XRD pattern of N/P CDs.

**Figure S3.** Relative fluorescence intensities of N/P CDs (10 *μ*g·mL^-1^) with varying NaCl concentrations in HEPES buffer (10 mM, pH 7.4).

**Figure S4.** The effect of temperature on relative fluorescence intensities of N/P CDs (10 *μ*g·mL^-1^).

**Figure S5.** Fluorescence intensity changes of N/P CDs (10 *μ*g·mL^−1^) for 120 min under 320 nm excitation with data collection once every 10 min.

**Figure S6.** (A) Relative fluorescence intensities of N/P CDs (10 *μ*g·mL^−1^) toward other metal ions (50 *μ*M) with and without Fe^3+^ (40 *μ*M). (B) Relative fluorescence intensities of N/P CDs (10 *μ*g·mL^−1^) + Fe^3+^ (40 *μ*M) toward other anions (50 *μ*M) in the absence and presence of F^−^ (30 *μ*M).

**Figure S7.** The fluorescence intensity of N/P CDs (10 *μ*g·mL^−1^) with and without Fe^3+^ (40 *μ*M) and F^−^ (30 *μ*M) at different pH values.

**Figure S8.** (A) Time-dependent relative fluorescent emission of N/P CDs (10 *μ*g·mL^−1^) for Fe^3+^ with various concentrations. (B) Time-dependent relative fluorescent emission of N/P CDs (10 *μ*g·mL^−1^) − Fe^3+^ (30 *μ*M) system for F^−^ with different concentrations.

**Figure S9.** Fluorescent intensity of N/P CDs (10 *μ*g·mL^-1^) at 392 nm in the HEPES buffer (10 mM, pH = 7.4) upon the alternate addition of Fe^3+^ (40 *μ*M) and F^−^ (30 *μ*M).

**Figure S10.** (A) Relative fluorescence intensities of N/P CDs (10 *μ*g·mL^−1^) toward other anions (50 *μ*M) in the absence and presence of MnO_4_^−^ (50 *μ*M). (B) Time-dependent relative fluorescent emission of N/P CDs (10 *μ*g·mL^−1^) for different concentrations of MnO_4_^−^. (C) The fluorescence intensity of N/P CDs (10 *μ*g·mL^−1^) with and without MnO_4_^−^ (50 *μ*M) at different pH values.

**Figure S11.** (A) Absorption spectra of MnO_4_^−^, excitation and emission spectra of N/P CDs. (B) Fluorescence decay curves of N/P CDs (10 *μ*g·mL^-1^) in the absence and presence of MnO_4_^−^ (50 *μ*M).

**Figure S12.** Fluorescence images of E. coli treated with CDs, and CDs + MnO_4_^−^. Images were captured with 488 (green) and 552 nm laser (red). The scale bar is 10 *μ*m.

**Table S1** The fluorescence quantum yields (QYs) of N/P CDs prepared with GMP under different hydrothermal conditions.

**Table S2** Comparison between the reported CDs and the prepared N/P CDs for Fe^3+^ detection.

**Table S3** Comparison of different fluorescent CDs based probes for F^−^ detection.

**Table S4** Comparison between the reported different CDs-based fluorescent sensors and the prepared N/P CDs for MnO_4_^−^ detection.

**Table S5** Detection of various analytes in lake water samples with N/P CDs.

**Experimental section**

**1. Materials**

Guanosine 5′-monophosphate disodium salt hydrate (GMP) was purchased from Macklin Biochemical Company (Shanghai, China). Quinine sulfate (99%, suitable for fluorescence) was supplied by Aladdin. All the metal salts (FeCl_3_·6H_2_O, KCl, CaCl_2_, BaCl_2_·2H_2_O, Hg(NO_3_)_2_·H_2_O, NaCl, MgSO_4_, CoCl_2_, FeSO_4_, LaCl_3_·χH_2_O, CuCl_2_·2H_2_O, MnSO_4_·H_2_O, AgNO_3_, ZnCl_2_, Cd(NO_3_)_2_·4H_2_O, Pb(NO_3_)_2_, Al(NO_3_)_3_·9H_2_O and Cr(NO_3_)_3_) and anionic salts (NaHCO_3_, KNO_3_, NaNO_2_, NaHSO_3_, NaClO_3_, Na_2_SO_4_, Na_2_SO_3_, K_2_CO_3_, NaF, K_2_S_2_O_8_, Na_3_PO_4_, Na_2_C_2_O_4_, NaCl, Na_2_HPO_4_, KI, H_3_BO_3_, KBr, and KMnO_4_) were obtained from Sinopharm Chemical Rea-gent Co., Ltd. (Shanghai, China). Ultrapure water was used throughout all the experiment.

**2. Apparatus**

UV-vis absorption spectra were recorded on a Varian Cary 300 spectrophotometer. The fluorescence spectra were recorded using a F-7000 spectrophotometer. Time-resolved fluorescence measurement was performed using a FM-4P-TCSPC spectrometer. The decay time was extracted by means of a re-convolution fit based on a triple exponential model. A Nicolet Nexus 870 FTIR spectrometer was used to record fourier transform infrared (FTIR) spectra, and samples were prepared in KBr pellets. A transmission electron microscopy (TEM, H-7650, Hitachi) was used to characterize the morphologies of the samples. Χ−ray photoelectron spectra (XPS) were recorded using a Thermo VG ESCALAB250 X-ray photoelectron spectrometer equipped with an Al Kα source. Powder X-ray diffraction (XRD) analyses were collected on a Rigaku D/max 2500V PC diffractometer with Cu Kα radiation. Bacteria fluorescent images were acquired by an upright optical microscope (Nikon Eclipse Ni-U).

**3. Preparation of N/P CDs**

Briefly, GMP (0.75 g) was dissolved in 30 mL ultrapure water and formed a clear solution under magnetic stirring. Then, this homogeneous solution was transferred to a 50 mL Teflon-lined autoclave and kept at 220 °C for 6 h. After the reaction, the brown solution was filtrated through a filtration membrane with pore size of 0.22 *μ*m to remove the large precipitates and then purified through a dialysis bag (1000 Da) for 48 h. The resulting solution was concentrated to be about 30 mL by rotary evaporation, and then obtained solid powder after vacuum freeze-drying for 48 h. Finally, the purified N/P CDs was dispersed in purified water (1.0 mg·mL^−1^) and stored at 4 °C for further characterization and use.

**4. Quantum yield (QY) measurement**

The QY of N/P CDs was determined according to a previously described method using quinine sulfate (QY_R_ = 54.4% in 0.10 M H_2_SO_4_ solution) as the reference standard (Nandi et al., 2021). In order to minimize the influence of reabsorption, the concentrations of all the samples were adjusted with the absorption below 0.05 at the excitation wavelength at 320 nm. And their fluorescence spectra were recorded under the same excitation wavelength at 320 nm. The QY of the obtained CDs was estimated according to the equation:


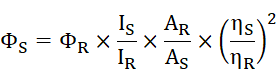


Where “R” and “S” refer to reference (quinine sulfate) and samples (CDs), respectively. “Φ” is the measuring QY of CDs, “I” is the integrated emission intensity, “A” is the the optical density and “η” is the refractive index (1.33 for water).

**5. Analysis of analyte in real samples**

To estimate the application in the practical analysis for environmental samples, the standard addition method was used to detect analyte in the lake water. The lake water sample was obtained from a local lake. The real samples were collected, centrifuged at 10 000 rpm for 10 minutes, and filtered through a membrane with pore size of 0.45 *μ*m to remove the insoluble substances. Then, different concentrations of analyte were added into the pretreated real samples. The fluorescent probe solution for analyte detection was used. The data were collected using a luminescence spectrometer. The average was obtained from three independent experiments, and a standard deviation was calculated.

**6. Cytotoxicity assays**

HeLa cells were purchased from the Cell Bank (Cell Institute, Sinica Academica Shanghai, Shanghai, China) and maintained in Dulbecco’s Modified Eagle’s Medium (DMEM) supplemented with 10% (v/v) fetal bovine serum (FBS) and 1% antibiotic solution at 37 °C in a 5% CO_2_ incubator. The cytotoxicity of N/P CDs was studied using HeLa cell lines by the standard MTT assay (Mukhtar-Fayyad et al., 2011). HeLa cells with a density of 1 × 10^4^ cells per well were seeded into a 96-well plate for 24 h, and then treated with different concentrations of tested CDs from 0 to 200 *μ*g·L^-1^ at 37 °C for another 24 h. At the end of the incubation, the MTT solution (20 *μ*L, 5 mg·mL^-1^) was mixed with each well and incubated for 4 h, and the absorbance at 570 nm was measured by a microplate reader (Bio-rad680, USA). Three tests were performed and expressed as mean ± standard deviation.


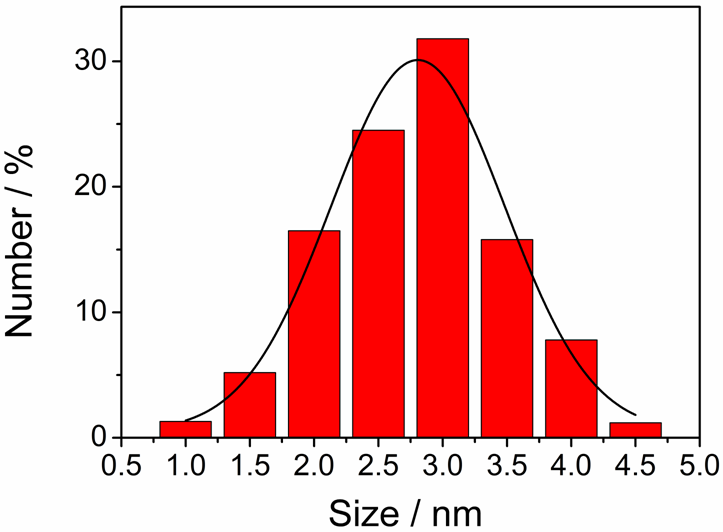


**Figure S1.** Size distribution of N/P CDs.


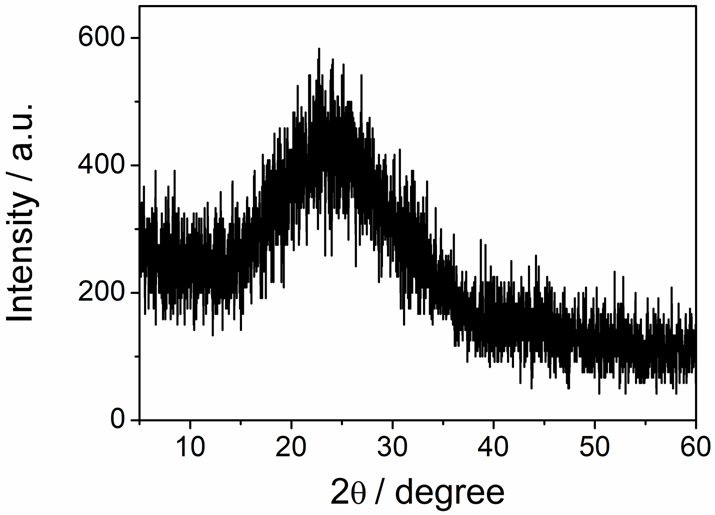


**Figure S2.** XRD pattern of N/P CDs.


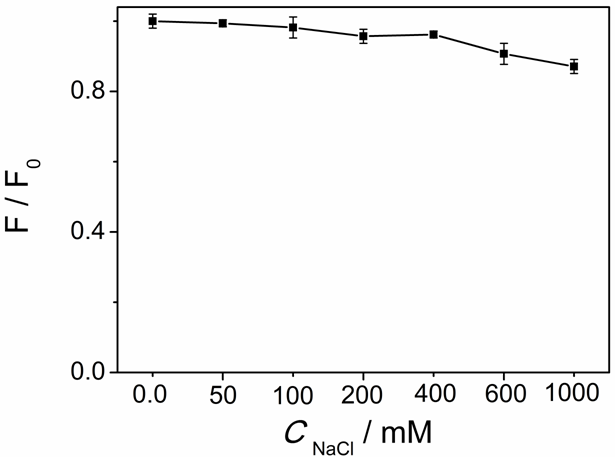


**Figure S3.** Relative fluorescence intensities of N/P CDs (10 *μ*g·mL^-1^) with varying NaCl concentrations in HEPES buffer (10 mM, pH 7.4).


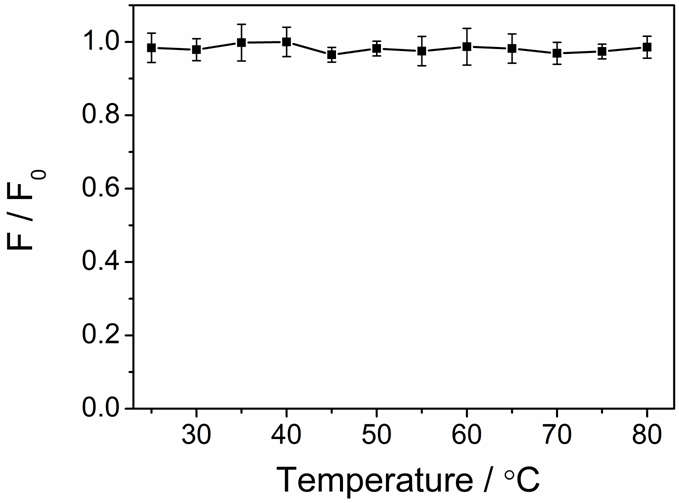


**Figure S4.** The effect of temperature on relative fluorescence intensities of N/P CDs (10 *μ*g·mL^-1^).


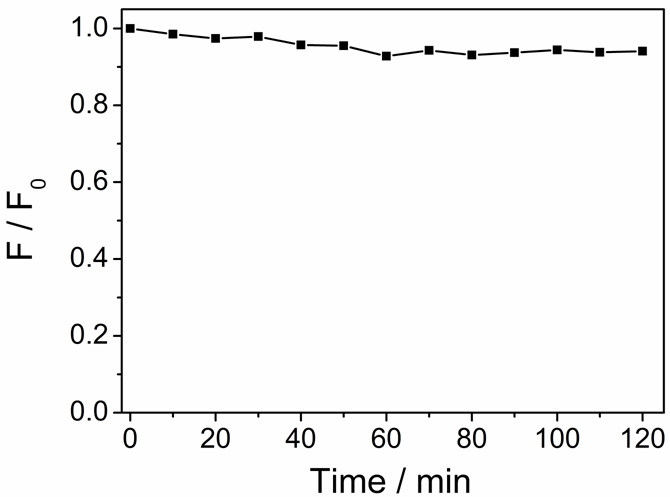


**Figure S5.** Fluorescence intensity changes of N/P CDs (10 *μ*g·mL^−1^) for 120 min under 320 nm excitation with data collection once every 10 min.


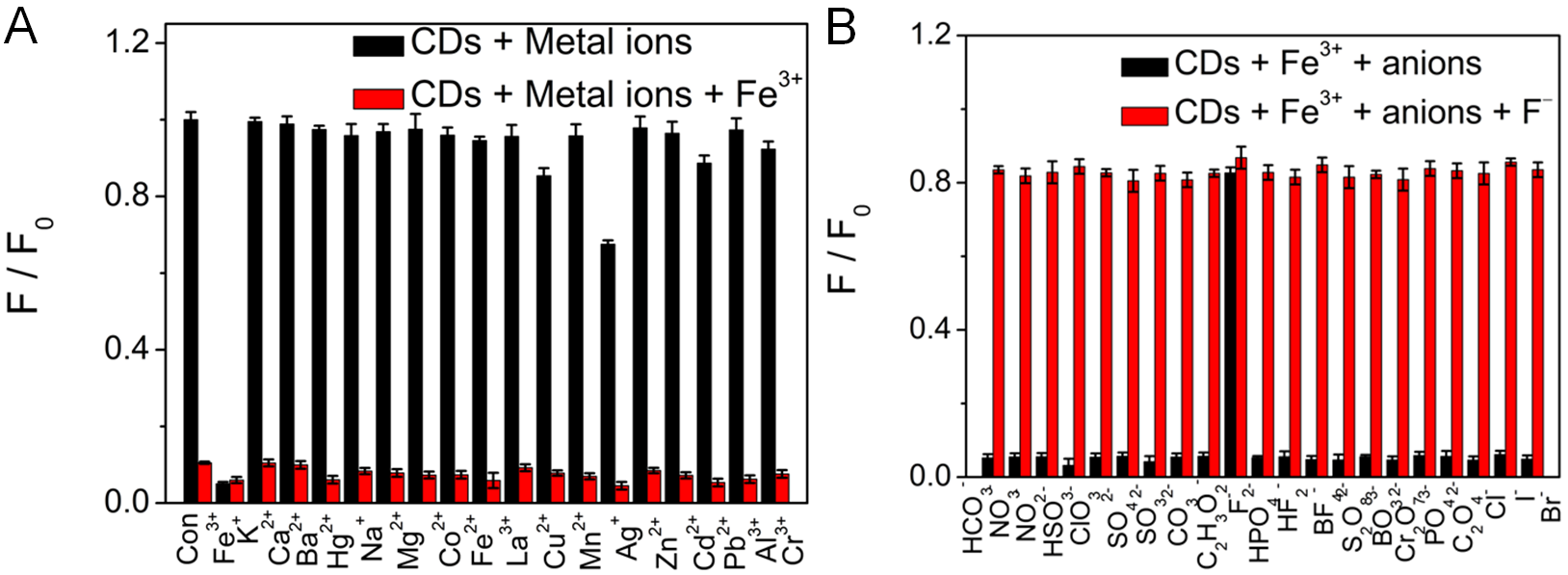


**Figure S6.** (A) Relative fluorescence intensities of N/P CDs (10 *μ*g·mL^−1^) toward other metal ions (50 *μ*M) with and without Fe^3+^ (40 *μ*M). (B) Relative fluorescence intensities of N/P CDs (10 *μ*g·mL^−1^) + Fe^3+^ (40 *μ*M) toward other anions (50 *μ*M) in the absence and presence of F^−^ (30 *μ*M).


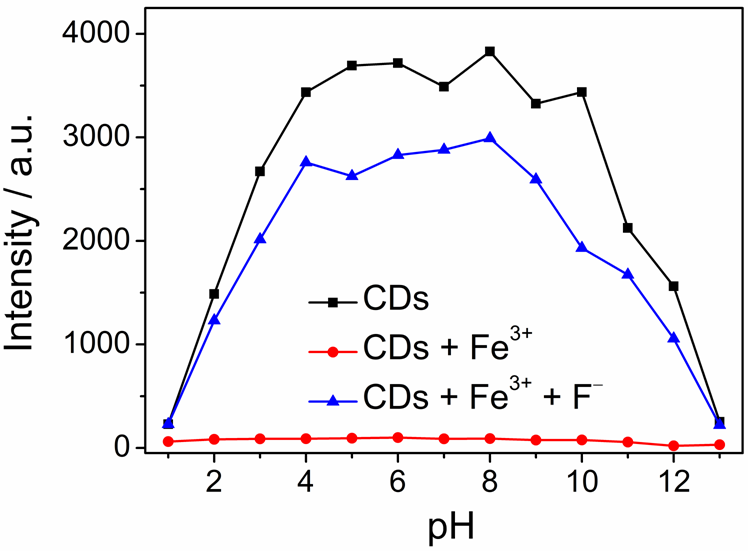


**Figure S7.** The fluorescence intensity of N/P CDs (10 *μ*g·mL^−1^) with and without Fe^3+^ (40 *μ*M) and F^−^ (30 *μ*M) at different pH values.


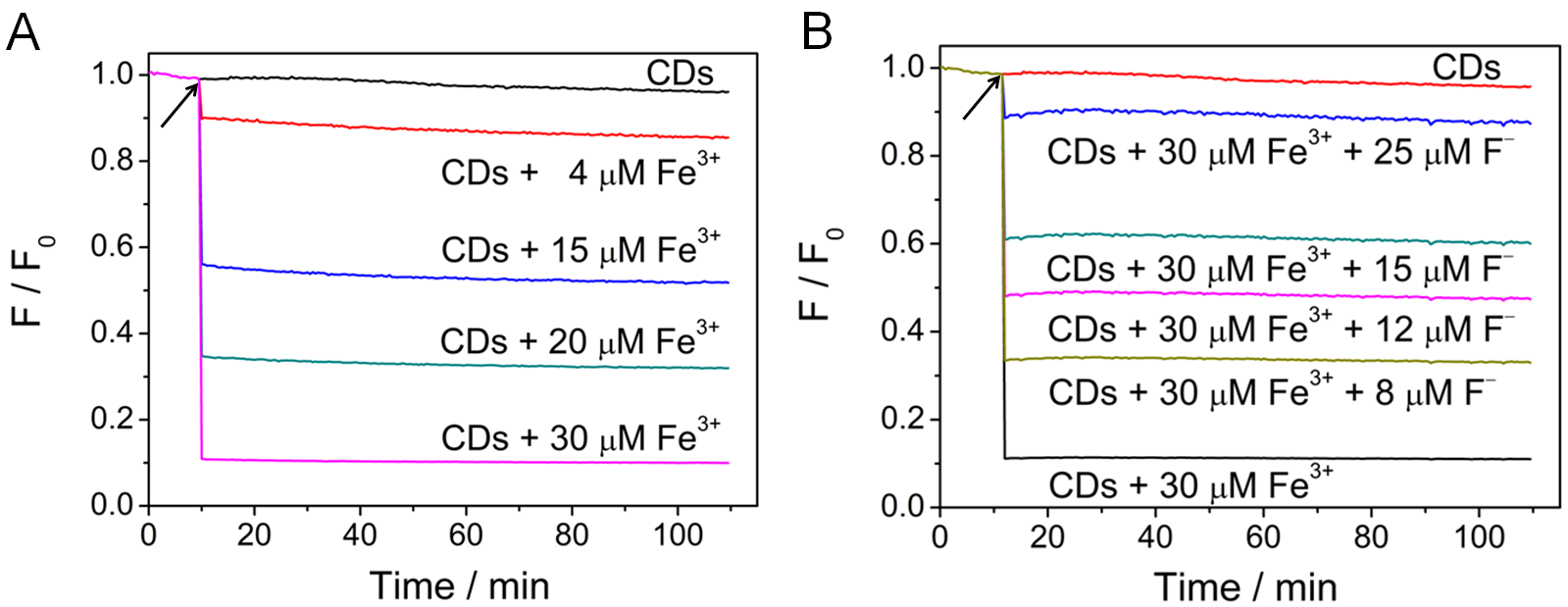


**Figure S8.** (A) Time-dependent relative fluorescent emission of N/P CDs (10 *μ*g·mL^−1^) for Fe^3+^ with various concentrations. (B) Time-dependent relative fluorescent emission of N/P CDs (10 *μ*g·mL^−1^) − Fe^3+^ (30 *μ*M) system for F^−^ with different concentrations.


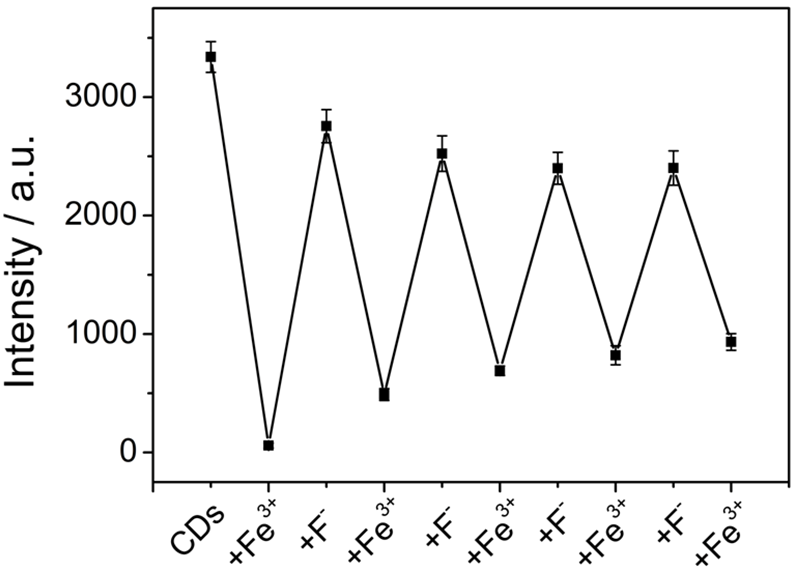


**Figure S9.** Fluorescent intensity of N/P CDs (10 *μ*g·mL^-1^) at 392 nm in the HEPES buffer (10 mM, pH = 7.4) upon the alternate addition of Fe^3+^ (40 *μ*M) and F^−^ (30 *μ*M).


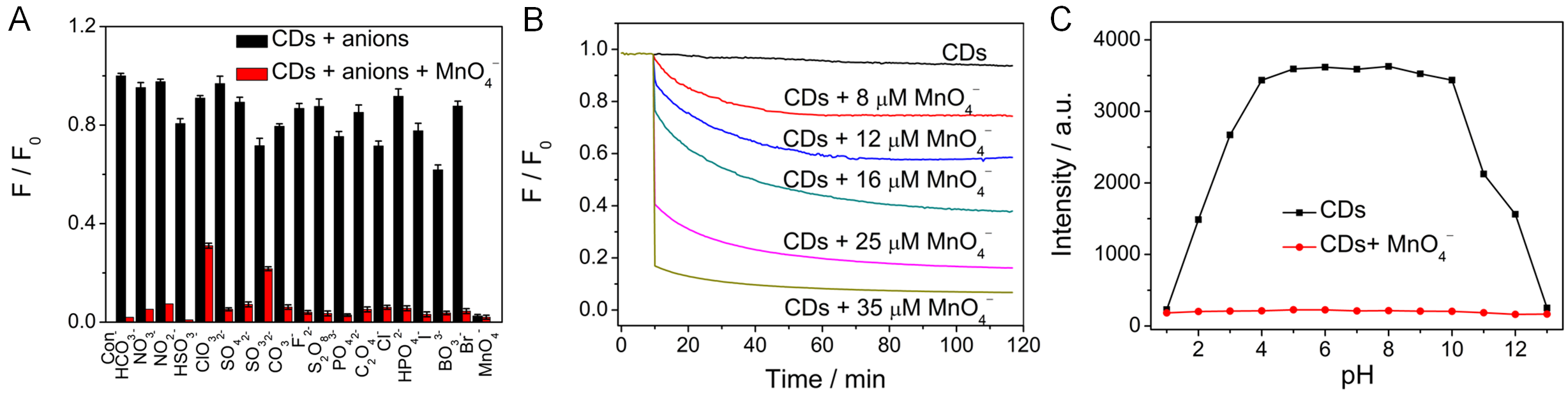


**Figure S10.** (A) Relative fluorescence intensities of N/P CDs (10 *μ*g·mL^−1^) toward other anions (50 *μ*M) in the absence and presence of MnO_4_^−^ (50 *μ*M). (B) Time-dependent relative fluorescent emission of N/P CDs (10 *μ*g·mL^−1^) for different concentrations of MnO_4_^−^. (C) The fluorescence intensity of N/P CDs (10 *μ*g·mL^−1^) with and without MnO_4_^−^ (50 *μ*M) at different pH values.


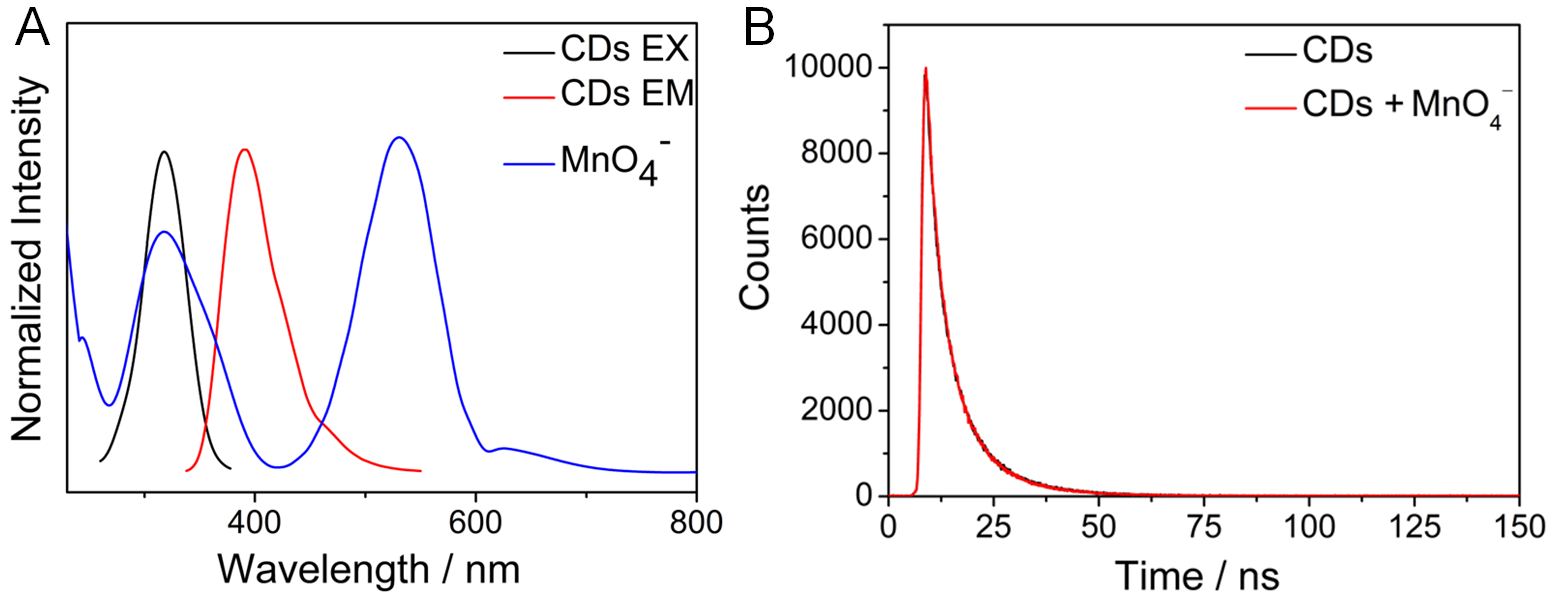


**Figure S11.** (A) Absorption spectra of MnO_4_^−^, excitation and emission spectra of N/P CDs. (B) Fluorescence decay curves of N/P CDs (10 *μ*g·mL^-1^) in the absence and presence of MnO_4_^−^ (50 *μ*M).


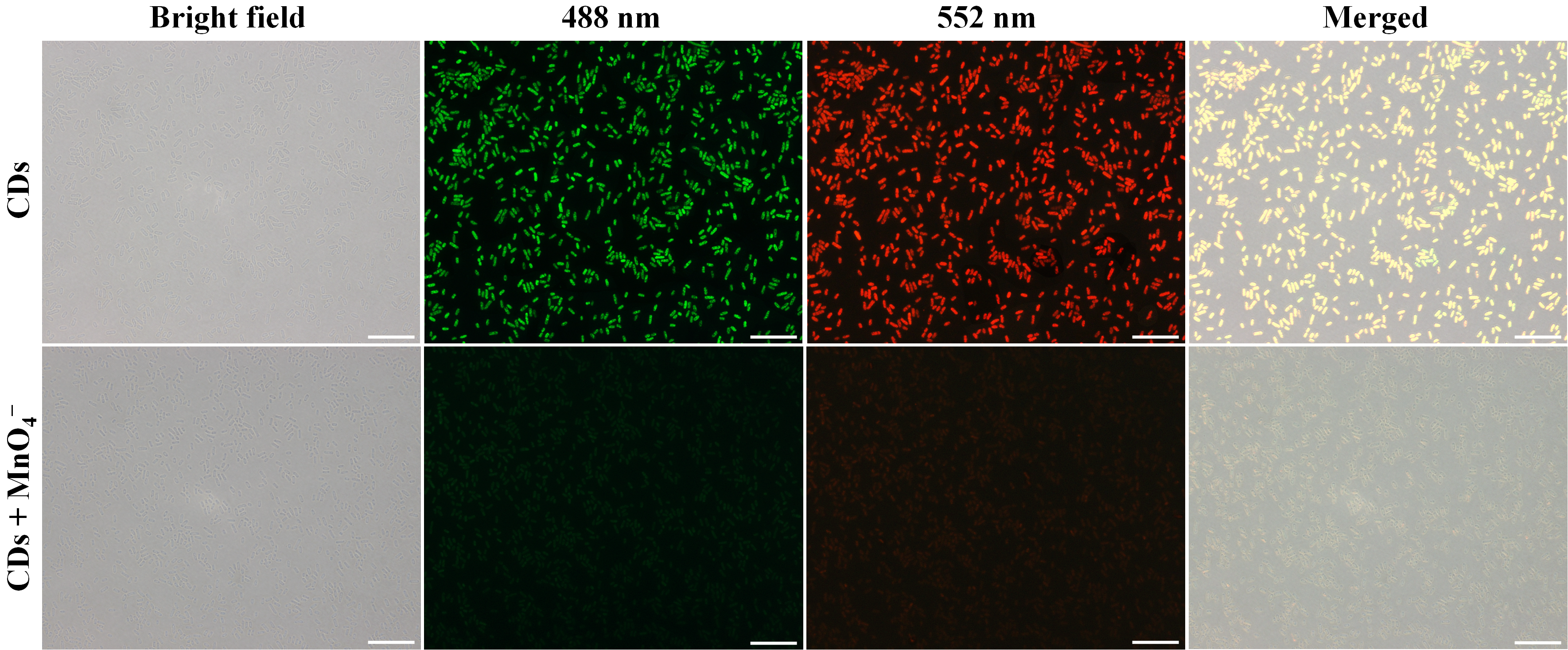


**Figure S12.** Fluorescence images of E. coli treated with CDs, and CDs + MnO_4_^−^. Images were captured with 488 (green) and 552 nm laser (red). The scale bar is 10 *μ*m.

**Table S1** The fluorescence quantum yields (QYs) of N/P CDs prepared with GMP under different hydrothermal conditions.

| Temperature / ^o^C | Time (h) | QY (%) |
| --- | --- | --- |
| 160 | 4 | 7.88 |
| 160 | 6 | 7.59 |
| 160 | 8 | 7.92 |
| 160 | 10 | 8.21 |
| 180 | 4 | 7.95 |
| 180 | 6 | 12.28 |
| 180 | 8 | 18.02 |
| 180 | 10 | 7.48 |
| 220 | 4 | 40.72 |
| 220 | 6 | 53.72 |
| 220 | 8 | 47.89 |
| 220 | 10 | 40.68 |

**Table S2** Comparison between the reported CDs and the prepared N/P CDs for Fe^3+^ detection.

| Kinds of CDs | QYs | LOD | ref |
| --- | --- | --- | --- |
| CDs | 9.8% | 15.4 nM | (Zhang et al., 2019) |
| CDs | 8.64% | 0.32 μM | (Shen et al., 2017) |
| CDs | 10.5% | 0.2 μM | (Sun et al., 2016) |
| CDs | 6.2% | 128 nM | (Jayaweera et al., 2019) |
| CDs | 10.85% | 9.55 μM | (Zulfajri et al., 2019) |
| CDs | 10.3% | 63.4 nM | (Rajendran et al., 2021) |
| N-CDs | 14% | 0.9 μM | (Atchudana et al., 2018) |
| Si-CDs | 27.2% | 13.7 nM | (Shamsipur et al., 2018) |
| N-CQDs | 23.48% | 0.75 μM | (Qi et al., 2019) |
| N-CDs | 54% | 42 nM | (Li et al., 2020) |
| CQDs | 15% | 50 nM | (Omer et al., 2018) |
| BNS-CDs | 5.44% | 90 nM | (Liu et al., 2017) |
| N,Zn-CDs | 63.28% | 27 nM | (Tammina et al., 2019) |
| N,S-CDs | 28.9% | 0.16 μM | (Chan et al., 2019) |
| N/S-CDs | 26% | 0.19 μM | (Liu et al., 2021) |
| N/P-CDs | 43.2% | 0.33 μM | (Shangguan et al., 2017) |
| N/P-CDs | 53.72% | 12 nM | This work |

**Table S3** Comparison of different fluorescent CDs based probes for F^−^ detection.

| Kinds of CDs | Linear range | LOD | ref |
| --- | --- | --- | --- |
| Fe_3_O_4_@SiO_2_@CDs | 1−20 μM | 60 nM | (Mohapatra et al., 2015) |
| *N*-CDs | 0−50 μM | 59 nM | (Zhang et al., 2021) |
| CDs-PyB | 0−200 μM | 59 μM | (Li et al., 2020) |
| CDs | 0−1.5 mM | 49 μM | (Liu et al., 2021) |
| FSMN/NCDs | 1−25 μM | 65 nM | (Li et al., 2021) |
| N/P-CDs | 0−20 μM | 8.5 nM | This work |

**Table S4** Comparison between the reported different CDs-based fluorescent sensors and the prepared N/P CDs for MnO_4_^−^ detection.

| Fluorescence probes | Linear range | LOD | ref |
| --- | --- | --- | --- |
|  |  |  |  |
| N/S-CDs |  |  | (Ding et al., 2020) |
|  | 1−20 μM | 0.34 μM |  |
|  |  |  |  |
|  |  |  |  |
| RhB-CDs@Cu-bca | 0−110 μM | 80 nM | (Zhu et al., 2019) |
| CDs@MOF(Eu) hybrid material | 0−100 μM | 0.68 μM | (Fu et al., 2018) |
|  |  |  |  |
|  |  |  |  |
|  |  |  |  |
|  |  |  |  |
|  |  |  |  |
|  |  |  |  |
|  |  |  |  |
| N/P-CDs |  |  | This work |
|  |  |  |  |
|  | 0−20 μM | 18.2 nM |  |

**Table S5** Detection of various analytes in lake water samples with N/P CDs.

| Sample | Spiked / μM | Found / μM | Recovery / % | RSD / % |
| --- | --- | --- | --- | --- |
| Fe^3+^ | 0 | Not detected | − | − |
|  | 1 | 1.13 | 102.9 | 1.08 |
|  | 5 | 5.23 | 100.12 | 1.05 |
|  | 10 | 9.62 | 99.92 | 0.94 |
| F^−^ | 0 | Not detected | − | − |
|  | 5 | 4.87 | 97.6 | 1.07 |
|  | 10 | 11.23 | 100.83 | 0.83 |
|  | 15 | 15.89 | 98.67 | 1.06 |
| MnO_4_^−^ | 0 | Not detected | − | − |
|  | 5 | 4.73 | 99.28 | 0.92 |
|  | 10 | 10.28 | 101.45 | 1.09 |
|  | 15 | 15.35 | 100.75 | 1.13 |

**References**

Atchudana, R., Edison, T. N. J. I., Aseer, K. R., Perumalc, S., Karthik, N., and Lee, Y. R. (2018). Highly fluorescent nitrogen-doped carbon dots derived from phyllanthus acidus utilized as a fluorescent probe for label-free selective detection of Fe^3+^ ions, live cell imaging and fluorescent ink. *Biosens. Bioelectron.* 99, 303−311.

Chan, K. K., Yang, C. B., Chien, Y. H., Panwar, N., and Yong, K. T. (2019). A facile synthesis of label-free carbon dots with unique selectivity-tunable characteristics for ferric ion detection and cellular imaging applications. *New J. Chem.* 43, 4734−4744.

Ding, C., Deng, Z., Chen, J., and Jin, Y. (2020). One-step microwave synthesis of N, S co-doped carbon dots from 1,6-hexanediamine dihydrochloride for cell imaging and ion detection. *Colloids Surf. B* 189, 110838.

Fu, X., Lv, R., Su, J., Li, H., Yang, B. Y., Gu, W., et al. (2018). A dual-emission nano-rod MOF equipped with carbon dots for visual detection of doxycycline and sensitive sensing of MnO_4_^−^. *RSC Adv*. 8, 4766–4772.

Jayaweera, S., Ke, Y., Hu, X., and Ng, W. J. (2019). Facile preparation of fluorescent carbon dots for label-free detection of Fe^3+^. *J. Photoch. Photobio. A* 370, 156−163.

Li, L., Shi, L. H., Jia, J., Chang, D., Dong, C., and Shuang, S. M. (2020). Fe^3+^ detection, bioimaging, and patterning based on bright blue-fluorescent N-doped carbon dots. *Analyst* 145, 5450−5457.

Li, M., Li, X. N., Jiang, M. Y., Liu, X., Chen, Z. J., Wang, S., et al. (2020). Engineering a ratiometric fluorescent sensor membrane containing carbon dots for efficient fluoride detection and removal. *Chem. Eng. J.* 399, 125741.

Li, X. L., Lin, H., Li, Q. L., Xue, J. Y., Xu, Y., and Zhuang, L. (2021). Recyclable magnetic fluorescent Fe_3_O_4_@SiO_2_ core−shell nanoparticles decorated with carbon dots for fluoride ion removal. *ACS Appl. Nano Mater.* 4, 3062−3074.

Liu, S. H., Liu, Z. C., Li, Q. F., Xia, H. J., Yang, W. J., Tian, B. S., et al. (2021). Facile synthesis of carbon dots from wheat straw for colorimetric and fluorescent detection of fluoride and cellular imaging. *Spectrochim. Acta A Mol. Biomol. Spectrosc.* 246, 118964.

Liu, Y. H., Duan, W. X., Song, W., Liu, J. J., Ren, C. L., Wu, J., et al. (2017). Red emission B, N, S-co-doped carbon dots for colorimetric and fluorescent dual mode detection of Fe^3+^ ions in complex biological fluids and living cells. *ACS Appl. Mater. Interfaces* 9, 12663−12672.

Liu, Q. L., Niu, X. Y., Xie, K. X., Yan, Y. M., Ren, B. R., Liu, R. R., et al. (2021). Fluorescent carbon dots as nanosensors for monitoring and imaging Fe^3+^ and [HPO_4_]^2-^ ions. *ACS Appl. Nano Mater.* 4, 190−197.

Mohapatra, S., Sahu, S., Nayak, S., and Ghosh, S. K. (2015) Design of Fe3O4@ SiO2@ carbon quantum dot based nanostructure for fluorescence sensing, magnetic separation, and live cell imaging of fluoride ion. *Langmuir* 31 (29), 8111–8120.

Mukhtar-Fayyad, D. (2011). Cytocompatibility of new bioceramic-based materials on human fibroblast cells (MRC-5). *Oral. Surg. Oral. Med. Oral. Pathol. Oral. Radiol. Endod*. 112, e137–e142.

Nandi, N., Gaurav, S., Sarkar, P., Kumar, S., and Sahu, K. (2021). Multifunctional N‑doped carbon dots for bimodal detection of Bilirubin and Vitamin B12, living cell imaging, and fluorescent ink. *ACS Appl. Bio Mater.* 4, 5201−5211.

Omer, K. M., Tofiq, D. I., and Hassan, A. Q. (2018). Solvothermal synthesis of phosphorus and nitrogen doped carbon quantum dots as a fluorescent probe for iron (III). *Mikrochim Acta*. 185, 466.

Qi, H. J., Teng, M., Liu, M., Liu, S. X., Li, J., Yu, H. P., et al. (2019). Biomass-derived nitrogen−doped carbon quantum dots: highly selective fluorescent probe for detecting Fe^3+^ ions and tetracyclines. *J. Colloid Interf. Sci.* 539, 332−341.

Rajendran, S., Ramanaiah, D. V., Kundu, S., and Bhunia, S. K. (2021). Yellow fluorescent carbon dots for selective recognition of As^3+^ and Fe^3+^ ions. *ACS Appl. Nano Mater.* 4, 10931−10942.

Shamsipur, M., Molaei, K., Molaabasi, F., Alipour, M., Alizadeh, N., Hosseinkhani, S., et al. (2018). Facile preparation and characterization of new green emitting carbon dots for sensitive and selective off/on detection of Fe^3+^ ion and ascorbic acid in water and urine samples and intracellular imaging in living cells. *Talanta* 183, 122−130.

Shangguan, J., Huang, J., He, D., He, X., Wang, K., Ye, R., et al. (2017). Highly Fe^3+^ selective fluorescent nanoprobe based on ultrabright N/P co-doped carbon dots and its application in biological samples. *Anal. Chem.* 89, 7477–7484.

Shen, J., Shang, S. M., Chen, X. Y., Wang, D., and Cai, Y. (2017). Facile synthesis of fluorescence carbon dots from sweet potato for Fe^3+^ sensing and cell imaging. *Mater. Sci. Eng. C* 76, 856−864.

Sun, C., Zhang, Y., Wang, P., Yang, Y., Wang, Y., Xu, J., et al. (2016). Synthesis of nitrogen and sulfur co-doped carbon dots from garlic for selective detection of Fe^3+^. *Nanoscale Res. Lett.*11, 1−9.

Tammina, S. K., Yang, D. Z., Li, X., Koppala, S., and Yang, Y. L. (2019). High photoluminescent nitrogen and zinc doped carbon dots for sensing Fe^3+^ ions and temperature. *Spectrochim. Acta A* 222,117141.

Zhang, W. Y., Jia, L. H., Guo, X. F., Yang, R., Zhang, Y., and Zhao, Z. L. (2019). Green synthesis of up- and down-conversion photoluminescent carbon dots from coffee beans for Fe^3+^ detection and cell imaging. *Analyst* 144, 7421−7431.

Zhang, Z. Y., Chen, X. Y., and Wang, J. L. (2021). Bright blue emissions N-doped carbon dots from a single precursor and their application in the trace detection of Fe^3+^ and F^-^. *Inorganica Chim. Acta.* 515, 120087.

Zhu, K., Fan, R. Q., Zheng, X. B., Wang, P., Chen, W., Sun, T. C., et al. (2019). Dual-emitting dye-CDs@MOFs for selective and sensitive identification of antibiotics and MnO_4_^−^ in water. *J. Mater. Chem.* *C* 7, 15057−15065.

Zulfajri, M., Gedda, G., Chang, C. J., Chang, Y. P., and Huang, G. G. (2019). Cranberry beans derived carbon dots as a potential fluorescence sensor for selective detection of Fe^3+^ ions in aqueous solution. *ACS Omega* 4, 15382–15392.
